# Relationship between sex, *APOE* genotype, endocannabinoids and cognitive change in older adults with metabolic syndrome during a 3-year Mediterranean diet intervention

**DOI:** 10.1186/s12937-024-00966-w

**Published:** 2024-06-12

**Authors:** Natalia Soldevila-Domenech, Beatriz Fagundo, Aida Cuenca-Royo, Laura Forcano, Maria Gomis-González, Anna Boronat, Antoni Pastor, Olga Castañer, Maria Dolores Zomeño, Albert Goday, Mara Dierssen, Khashayar Baghizadeh Hosseini, Emilio Ros, Dolores Corella, Miguel Ángel Martínez-González, Jordi Salas-Salvadó, Fernando Fernández-Aranda, Montserrat Fitó, Rafael de la Torre

**Affiliations:** 1 Integrative Pharmacology and Systems Neurosciences Research Group, Neurosciences Research Program, Hospital del Mar Research Institute (HMRI), Barcelona, 08003 Spain; 2 Department of Medicine and Life Sciences, Universitat Pompeu Fabra, Barcelona, 08003 Spain; 3 Department of Physiotherapy, Fundació Universitària del Bages (FUB), Manresa, 08042 Spain; 4 CIBER de Fisiopatología de la Obesidad y Nutrición, Instituto de Salud Carlos III, Madrid, 28029 Spain; 5 Cardiovascular Risk and Nutrition Research Group, Hospital del Mar Research Institute (HMRI), Barcelona, 08003 Spain; 6 Endocrinology Service, Hospital del Mar Research Institute (HMRI), Barcelona, 08003 Spain; 7 School of Health Sciences, Blanquerna-Ramon Llull University, Barcelona, 08022 Spain; 8 Centre for Genomic Regulation, The Barcelona Institute of Science and Technology, Barcelona, 08003 Spain; 9 CIBER de Enfermedades Raras (CIBERER), Barcelona, Spain; 10 Cardiovascular risk, Nutrition and Aging, Institut d’Investigacions Biomèdiques August Pi Sunyer (IDIBAPS), Hospital Clínic, Barcelona, 08036 Spain; 11 Department of Preventive Medicine and Public Health, School of Medicine, University of Valencia, Valencia, 46010 Spain; 12 Department of Preventive Medicine and Public Health, University of Navarra, Pamplona, Spain; 13 Navarra’s Health Research Institute (IdiSNA), Pamplona, Spain; 14 Departament de Bioquímica i Biotecnologia, Universitat Rovira i Virgili, Unitat de Nutrició Humana, Reus, Spain; 15 Institut d’Investigació Sanitària Pere Virgili (IISPV), Reus, Spain; 16 Clinical Psychology Unit, University Hospital of Bellvitge-IDIBELL, L’Hospitalet de Llobregat, Barcelona, 08908 Spain; 17 Department of Clinical Sciences, School of Medicine and Health Sciences, University of Barcelona, Barcelona, Spain; 18 Psychoneurobiology of Eating and Addictive Behaviors Group, Neuroscience Program, Institut d’Investigació Biomèdica de Bellvitge-IDIBELL, L’Hospitalet de Llobregat, Barcelona, 08908 Spain; 19 Neurosciences Research Program, Hospital del Mar Research Institute (HMRI), Dr Aiguader 88, Barcelona, 08003 Spain

**Keywords:** Mediterranean diet, Endocannabinoids, Sex differences, Cognition, Metabolic syndrome, 2-AG

## Abstract

**Background:**

The Mediterranean diet (MedDiet) has demonstrated efficacy in preventing age-related cognitive decline and modulating plasma concentrations of endocannabinoids (eCBs) and *N-*acylethanolamines (NAEs, or eCB-like compounds), which are lipid mediators involved in multiple neurological disorders and metabolic processes. Hypothesizing that eCBs and NAEs will be biomarkers of a MedDiet intervention and will be related to the cognitive response, we investigated this relationship according to sex and apolipoprotein E (*APOE)* genotype, which may affect eCBs and cognitive performance.

**Methods:**

This was a prospective cohort study of 102 participants (53.9% women, 18.8% *APOE*-ɛ4 carriers, aged 65.6 ± 4.5 years) from the PREDIMED-Plus-*Cognition* substudy, who were recruited at the Hospital del Mar Research Institute (Barcelona). All of them presented metabolic syndrome plus overweight/obesity (inclusion criteria of the PREDIMED-Plus) and normal cognitive performance at baseline (inclusion criteria of this substudy). A comprehensive battery of neuropsychological tests was administered at baseline and after 1 and 3 years. Plasma concentrations of eCBs and NAEs, including 2-arachidonoylglycerol (2-AG), anandamide (AEA), oleoylethanolamide (OEA), palmitoylethanolamide (PEA), and *N-*docosahexaenoylethanolamine (DHEA), were also monitored. Baseline cognition, cognitive changes, and the association between eCBs/NAEs and cognition were evaluated according to gender (crude models), sex (adjusted models), and *APOE* genotype.

**Results:**

At baseline, men had better executive function and global cognition than women (the effect size of gender differences was − 0.49, *p* = 0.015; and − 0.42, *p* = 0.036); however, these differences became nonsignificant in models of sex differences. After 3 years of MedDiet intervention, participants exhibited modest improvements in memory and global cognition. However, greater memory changes were observed in men than in women (Cohen’s d of 0.40 vs. 0.25; *p* = 0.017). In men and *APOE*-ε4 carriers, 2-AG concentrations were inversely associated with baseline cognition and cognitive changes, while in women, cognitive changes were positively linked to changes in DHEA and the DHEA/AEA ratio. In men, changes in the OEA/AEA and OEA/PEA ratios were positively associated with cognitive changes.

**Conclusions:**

The MedDiet improved participants’ cognitive performance but the effect size was small and negatively influenced by female sex. Changes in 2-AG, DHEA, the OEA/AEA, the OEA/PEA and the DHEA/AEA ratios were associated with cognitive changes in a sex- and *APOE*-dependent fashion. These results support the modulation of the endocannabinoid system as a potential therapeutic approach to prevent cognitive decline in at-risk populations.

**Trial registration:**

ISRCTN89898870.

**Supplementary Information:**

The online version contains supplementary material available at 10.1186/s12937-024-00966-w.

## Background

Preventing age-related cognitive decline is paramount as the number of individuals older than 60 years old is increasing dramatically and age is the strongest risk factor for Alzheimer’s disease (AD) and related dementias [1, 2]. Adherence to a Mediterranean-like dietary pattern (MedDiet) has been associated with a decreased risk of cognitive decline and dementia [3, 4]. These beneficial effects could be attributed partially to improved lipid metabolism [5], as the pathogenesis of cognitive decline involves disturbances in lipid homeostasis, both in the brain and at a systemic level [6–8]. Moreover, the protein apolipoprotein E (APOE), which plays a critical role in lipid metabolism, is the most important genetic risk factor for late-onset AD, as carriers of the *APOE*-ɛ4 allele are at significantly higher risk of AD than carriers of the common *APOE*-ɛ3 allele [9, 10].

Lipid mediators are lipid-derived signaling molecules that regulate energy metabolism and systemic inflammation, in addition to other processes [7]. Among lipid mediators, endocannabinoids (eCBs) are implicated in multiple neurological disorders because they regulate brain development, whole-body homeostasis, neurotransmitter release, and synaptic plasticity [11, 12]. The most studied eCBs are 2-arachidonoyl-glycerol (2-AG) and anandamide (AEA). They are synthesized *on-demand* by cell membrane phospholipids, and can activate type-1 and type-2 cannabinoid receptors, in addition to other targets [11, 13]. The role of eCBs in cognitive processes has been extensively studied in animal models [11, 14]. However, evidence in humans is limited, with studies pointing to beneficial, detrimental, or null effects of eCBs in cognitively normal individuals [15, 16] or those with AD [17, 18]. Moreover, although plasma concentrations of eCBs are assumed to reflect their overall availability throughout the body, there is still controversy about whether they are reliable biomarkers of brain eCB signaling [19].

Short- and long-term MedDiet interventions have been shown to modulate plasma concentrations of eCBs and eCB-like molecules (*N-*acylethanolamines, NAEs) and their relative abundance in the form of ratios [20, 21]. Specifically, during the three years of MedDiet intervention, a persistent decrease in 2-AG was observed, which was strongly associated with triglyceride concentrations [21]. There were also reductions in AEA and other NAEs after six months, including oleoylethanolamide (OEA, derived from oleic acid), palmitoylethanolamide (PEA, derived from palmitic acid), and *N-*docosahexaenoylethanolamine (DHEA, derived from docosahexaenoic acid -DHA), while the OEA/PEA, OEA/AEA, and DHEA/AEA ratios increased after six months or one year of MedDiet intervention [21]. These changes in eCBs or NAEs were not affected by changes in their precursor fatty acids and were associated with changes in insulin resistance and the achievement of clinically meaningful weight loss [21].

Sex and *APOE* genotype have been identified as determinants of eCBs concentrations [21, 22] and may also affect the cognitive response to lifestyle interventions [23, 24]. On one hand, postmenopausal women with metabolic syndrome and overweight or obesity present elevated concentrations of AEA and its congeners, whereas men display higher ratios of OEA/AEA, DHEA/AEA, and PEA/AEA [21]. Moreover, women may be more resistant to changes in circulating NAEs after a MedDiet intervention and may also experience fewer cardiovascular and cognitive benefits than men [21, 23]. On the other hand, *APOE*-ɛ4 carriers exhibit alterations in eCBs and related lipids compared to noncarriers [22]. Accordingly, the presence of the *APOE*-ɛ4 genetic variant reduces the expression of the APOE-binding neuronal receptor sortilin, which controls cholesterol transport and facilitates the neuronal metabolism of polyunsaturated fatty acids to eCBs and NAEs [22]. Therefore, stratified analyses by sex and *APOE* genotype are necessary for elucidating the potential relationship between eCBs and cognitive changes, as recently proposed by others [25–27].

Understanding how diet affects cognition and whether its effects differ according to nonmodifiable risk factors such as sex and *APOE* genotype is critical for informing targeted prevention strategies in at-risk populations [28]. We have previously shown that following the MedDiet can slow down age-related cognitive decline and promote improvements in memory, executive functions, and global cognition composites, as well as in the specific domains of visuospatial and verbal memory, visuoconstructive praxis and attention, and inhibition [23]. However, whether these benefits differ according to sex and *APOE* genotype is still unclear. Moreover, psychosocial and cultural factors such as age, educational level, socioeconomic status, mental health, or social interactions, could also influence the cognitive response to lifestyle interventions, and thus gender differences should be considered in addition to biological sex differences [29].

In this report, we hypothesize that gender, sex and *APOE* genotype will modulate the cognitive benefits of a MedDiet intervention and that eCBs in plasma will be biomarkers of these effects. Specifically, this study aimed (i) to analyze the influence of gender, sex and the *APOE*-ɛ4 genotype on cognitive changes ensuing from a MedDiet intervention; (ii) to evaluate the influence of the *APOE*-ɛ4 genotype on the modulation of eCBs, and cardiometabolic and lifestyle risk factors; and (iii) to examine the relationship between changes in eCBs and cognitive changes by sex and *APOE* genotype.

## Methods

### Study design and population

This prospective cohort study included 102 participants (55 women, 47 men) from the PREDIMED-Plus-*Cognition* substudy [23] who were recruited at the Hospital del Mar Research Institute (HMRI) study site (Barcelona, Spain), where additional blood samples were collected for determinations of eCBs [21]. The PREDIMED-Plus-*Cognition* is a substudy of the PREDIMED-Plus, in which a comprehensive neuropsychological evaluation was performed at baseline and after 1 and 3 years of intervention and involved the participation of four centers (HMRI, Barcelona, Spain; Rovira i Virgili University, Reus, Spain; University of Valencia, Valencia, Spain; and Bellvitge University Hospital, Barcelona, Spain) [23]. In the present study, participants were grouped into *APOE-*ε4 carriers (i.e., 1 or 2 *APOE-*ε4 alleles, *N* = 19) and *APOE*-ε4 noncarriers (*N* = 83). In contrast to the negative effect of *APOE-*ε4, the *APOE-*ε2 allele is protective against AD [30, 31]; thus, *APOE-*ε2ε4 participants (*N* = 3 out of 105) were excluded from further analyses.

The PREDIMED-Plus is a multicenter randomized parallel-group primary prevention trial (*N* = 6,874) that aims to evaluate the long-term effectiveness of a lifestyle intervention with an energy-reduced MedDiet (er-MedDiet, involving 30% calorie restriction), physical activity promotion and behavioral support with weight loss goals (intervention group), compared to a more traditional calorie-unrestricted MedDiet intervention without physical activity promotion or weight loss goals (‘active’ control group), on the long-term maintenance of weight loss and the prevention of cardiovascular disease [32–34]. Participants allocated to the traditional, calorie-unrestricted MedDiet group were instructed to progressively increase compliance with the 14-item MEDAS questionnaire [35]. Specifically, they were instructed to: (1) increase their consumption of vegetables (≥ 2 servings/day; 1 serving = 200 g), fruit (≥ 3 servings/day), nuts (≥ 3 servings/week; 1 serving = 30 g), and fish/seafood (≥ 3 servings/day; 1 serving = 100–150 g of fish, or 200 g of seafood); (2) use olive oil as the main culinary fat (≥ 4 tablespoon/day, 1 tablespoon = 13.5 g); (3) decrease their intake of red or processed meat (< 1 serving/day, 1 serving = 100–150 g); (4) prepare homemade traditional foods based on “sofrito” (a mixture of stir-fried tomato, onions, garlic, and aromatic herbs); and (5) in participants who reported drinking alcohol, moderate consumption of red wine (limited to 300 ml/day or 28 g/day of alcohol in men and 14 g/day in women) with meals was recommended. Participants in the energy-reduced MedDiet group received counseling to progressively increase compliance with the 17-item er-MEDAS questionnaire [36], with recommendations to progressively reach a 30% decrease in energy requirement according to each participant’s basal metabolic rate, resulting in a reduction of about 500 kcal/day. The main differences with the control group in specific recommendations included: <1 serving of red meat/week (instead of < 1 serving/day); <1 serving/week of butter instead of < 1 serving/day (1 serving = 12 g); and < 1 serving/week of sugar-sweetened beverages (instead of < 1 serving/day). Moreover, they were instructed to avoid the addition of sugar in tea/coffee, consume ≤ 1 serving/day of white bread (1 serving = 75 g), consume ≥ 5 servings/week of whole grain bread or whole grain pasta, and consume < 3 servings/week of refined bread, rice and/or pasta. To reinforce MedDiet adherence, participants in both arms of the trial received an allotment of extra-virgin olive oil (1 L/month), and at the beginning of the study received 125 g of raw almonds [32]. They were encouraged to consume 500 g/month of mixed nuts, including walnuts, peanuts, hazelnuts and almonds.

In the present study framed within the PREDIMED-Plus-*Cognition*, participants allocated to the intervention or control groups were pooled together and analyzed as a prospective cohort, as both groups were exposed to a MedDiet intervention and did not differ in cognitive induced changes over time [23]. They also showed minimal differences in the modulation of eCBs over the 3-year follow-up period [21].

The inclusion criteria of the PREDIMED-Plus study included community-dwelling overweight or obese individuals (body mass index (BMI) between 27 and 40 kg/m^2^), aged between 55 and 75 years for men and between 60 and 75 years for women, who met at least three criteria of the metabolic syndrome [37]. PREDIMED-Plus participants were invited to participate in the PREDIMED-Plus-*Cognition* substudy if they presented normal cognitive performance at baseline and did not meet the following exclusion criteria: (i) a history of chronic medical illness or neurological conditions that may affect cognitive function; (ii) a current psychiatric diagnosis or in a year prior to inclusion; (iii) traumatic brain injury with loss of consciousness of more than 2 minutes, learning disorders, or mental retardation; (iv) psychoactive substance abuse or dependence (either currently or in the past six months); and (v) a comorbid eating disorder [38].

The clinical trial was registered in the International Standard Randomized Controlled Trial database (ISRCTN; 89,898,870). All participants provided written informed consent prior any study related procedures. The study protocol was approved by the local institutional review board (Parc de Salut Mar Clinical Research Ethics Committee CEIm-PSMAR) and adheres to the standards of the WAMA Declaration of Helsinki (Brazil, October 2013).

### Variables

#### Sex and gender conceptualization

The term ‘gender’ refers to sociocultural norms, relationships, and identities that structure societies and shape behaviors, environments, and knowledge [39]. The term ‘sex’ refers to the biological and physical characteristics that define men and women. The binary variable of self-identified gender (men/women) was used in this study to investigate both gender and sex differences in cognitive performance. The gender effect represents the unadjusted, or crude, impact of this variable on outcomes, without accounting for any other variable, in order to reflect broader sociocultural influences. The sex effect, on the other hand, accounts for confounding by factors associated with gender and sex, in order to isolate, as far as possible, the influence of biological and physical characteristics [40]. To isolate the specific impact of sex, models were adjusted for factors linked to both sex/gender and cognition. These factors include age, education, diabetes, mental health (measured by tranquilizer or sedative use as a proxy), dyslipidemia (measured by lipid-lowering agent use as a proxy), lifestyle behaviors (including MedDiet adherence and physical activity), and *APOE* genotype [23].

#### Cognitive performance

Cognitive function was assessed at baseline and after 1 and 3 years by trained neuropsychologists and included the following domains: (i) *short-term and long-term auditory memory*, assessed with the Rey Auditory-Verbal Learning Test (RAVLT) [41, 42]; (ii) *short-term and long-term visuospatial memory, visual perception and visuoconstructive praxis and attention*, evaluated with the Rey-Osterrieth Complex Figure Test (ROCF) [43, 44]; (iii) *processing speed*, evaluated with the Symbol Digit Modalities Test (SDMT) [45]; (iv) *inhibition and attention* (mental flexibility and interference resistance) evaluated with the Stroop Color-Word Test [46]; (v) *decision-making abilities* evaluated with the Iowa Gambling Task (IGT) [47]; and (vi) *inattention, impulsivity, and vigilance* evaluated with the Conner’s Continuous Performance Test (CPT) [48]. Except for the CPT, higher scores on these neuropsychological tests indicate better performance. Finally, a baseline cognitive screening was also included using the Folstein Mini-Mental State Examination (MMSE) [49], for which scores greater than 24 were used to define normal cognitive function.

Primary cognitive outcomes comprised composite scores of memory, executive functions and global cognition. Composite scores were calculated for each participant by standardizing the raw test scores to z scores using the mean and standard deviation of the baseline data. The memory composite was created by averaging the z scores of the RAVLT immediate recall (RAVLT-IR) and delayed recall (RAVLT-DR) scores, and the ROCF immediate recall (ROCF-IR), delayed recall (ROCF-DR) and recognition (ROCF-R) scores. In turn, the executive functions composite was created by averaging the z scores of the ROCF figure copy (ROCF-C) score, the SDMT total score, the Stroop interference score, the IGT total score, and the reversed scores of the CPT omission and commission errors and hit reaction time (HRT) (higher scores indicate lower cognitive performance). The ROCF-C and the CPT omission error scores deviated from a normal distribution, prompting normalization through ordered quantile (ORQ) transformation [50]. Finally, the global cognition composite included the 12 scores of memory and executive functions. These cognitive composites have been used in previous studies [23, 51].

#### Lifestyle and cardiovascular risk factors

These parameters were measured 4 times: at baseline and after 6 months, 1 year and 3 years. Adherence to the er-MedDiet was evaluated by trained dietitians with the 17-item er-MEDAS questionnaire [36]. The values ranged from 0 to 17, with higher values indicating greater adherence. Leisure-time physical activity was measured as metabolic equivalent tasks (METs-minute/week) and evaluated with the Minnesota REGICOR Short Physical Activity Questionnaire (VREM) [52]. Anthropometric factors, including weight, height, and hip and waist circumferences, were measured by nurses via standardized procedures. Blood pressure was measured in triplicate using a validated semiautomatic oscillometer (Omron HEM 297 705 C).

Blood samples were collected after an overnight fast to determine lipid concentrations (triglycerides, total cholesterol and high-density lipoprotein cholesterol, HDL-c) and glycemic concentrations (glucose, glycosylated hemoglobin-HbA1c) using standard methodology. LDL cholesterol concentrations were calculated with the Friedewald formula whenever triglycerides were less than 300 mg/dL. Baseline type 2 diabetes was defined by a previous clinical diagnosis of diabetes, an HbA1c ≥ 6.5%, the use of antidiabetic medication or insulin, or a fasting plasma glucose > 126 mg/dL. Finally, insulin resistance was estimated using the homeostasis model assessment of insulin resistance (HOMA-IR) index [53].

#### APOE genotyping

Genomic DNA was extracted from buffy coat with the MagNaPure LC DNA Isolation Kit (Roche Diagnostics, Mannheim, Germany). A validated single-tube protocol using fluorescent probes in the LightTyper instrument (Roche) was used for *APOE* genotyping, as previously reported [54]. Quality control procedures including positive and negative controls as well as replication of a random 15% of samples were applied.

#### eCBs quantification

eCBs were quantified in plasma at baseline, and after 6 months, 1 year and 3 years by LC‒MS/MS following a previously validated method [55]. A specific pre-analytical treatment for the fresh blood samples is required within the first 30 min after blood collection [21]. The following compounds were quantified: 2-arachidonoylglycerol (2-AG), anandamide or *N*-arachidonoyl-ethanolamine (AEA), *N*-dihomo-γ-linolenoyl ethanolamide (DGLEA), *N*-docosatetraenoylethanolamine (DEA), *N*-docosahexaenoylethanolamine (DHEA), *N*-linoleoylethanolamine (LEA), oleoylethanolamide (OEA), *N*-palmitoleoylethanolamine (POEA), palmitoylethanolamide (PEA), and *N*-stearoylethanolamine (SEA). The ratios between OEA/AEA, PEA/AEA, DHEA/AEA and OEA/PEA were also calculated.

### Statistical analyses

Descriptive statistics for all variables at baseline are reported as mean and standard deviation (SD) or as the median and quartiles 1 and 3 (Q1, Q3), stratified by group (i.e., sex or *APOE* genotype). Descriptive statistics by sex/gender were focused solely on cognitive outcomes, since details on eCBs, cardiometabolic and lifestyle factors by sex have been published elsewhere [21]. Group differences at baseline were analyzed using Cohen’s d effect size [56] and linear regression models. Two different models were devised to evaluate gender and sex differences, respectively. Gender differences were examined through unadjusted linear regression models (i.e., crude models), reporting regression coefficients (β) with 95% confidence intervals (95%CI), adjusted R-squared (R^2^), and p-values. Sex differences in baseline cognitive performance were evaluated using linear regression models adjusted for gender-related factors (age, years of education, diabetes, use of tranquilizers or sedatives, use of lipid-lowering agents, baseline MedDiet adherence and baseline physical activity), and *APOE* genotype (referred to as ‘sex covariates’), as described in previous studies [23, 40]. Baseline differences between *APOE-*ε4 carriers and noncarriers were analyzed using linear models adjusted for gender, age, smoking status, and use of lipid-lowering agents (referred to as ‘*APOE* covariates’), as these were found to be unbalanced between groups (Table 1).

**Table 1:** Baseline characteristics of the study population stratified by sex and *APOE*-ε4 genotype

Variable		All	Sex differences			*APOE*- ε4 differences		
			Men	Women	*P*-value^*^	Noncarriers	Carriers	*P*-value^*^
	Category	*N* (%)	*N* (%)	*N* (%)		*N* (%)	*N* (%)	
N		102	47	55		83	19	
Sex	Women	55 (53.9)				48 (57.8)	7 (36.8)	0.161
*APOE*-ε4 carriers		19 (18.8)	12 (26.1)	7 (12.7)	0.146			
Intervention group		50 (49.0)	24 (51.1)	26 (47.3)	0.855	38 (45.8)	12 (63.2)	0.266
Age (years)	*Mean (SD)*	65.6 (4.51)	65.0 (5.19)	66.2 (3.80)	0.192	65.9 (4.52)	64.6 (4.45)	0.266
Education (years)	*Mean (SD)*	11.6 (4.13)	13.2 (4.37)	10.2 (3.40)	**< 0.001**	11.6 (4.08)	11.4 (4.46)	0.827
Education level	Primary	35 (34.3)	10 (21.3)	25 (45.5)	**0.028**	29 (34.9)	6 (31.6)	0.901
	Secondary	39 (38.2)	20 (42.6)	19 (34.5)		32 (38.6)	7 (36.8)	
	University	28 (27.5)	17 (36.2)	11 (20.0)		22 (26.5)	6 (31.6)	
Employment status	Employed	22 (21.6)	16 (34.0)	6 (10.9)	**0.005**	16 (19.3)	6 (31.6)	0.246
	Retired	72 (70.6)	30 (63.8)	42 (76.4)		59 (71.1)	13 (68.4)	
	Other	8 (7.84)	1 (2.13)	7 (12.7)		8 (9.64)	0 (0.00)	
Married		80 (78.4)	40 (85.1)	40 (72.7)	0.203	64 (77.1)	16 (84.2)	0.758
Current smoker		10 (9.80)	8 (17.0)	2 (3.64)	**0.041**	6 (7.23)	4 (21.1)	0.087
Type 2 diabetes		47 (46.1)	25 (53.2)	22 (40.0)	0.257	115 (36.2)	125 (26.0)	0.176
Obesity		86 (84.3)	39 (83.0)	47 (85.5)	0.944	36 (43.4)	11 (57.9)	0.373
MMSE score	*Mean (SD)*	28.7 (1.21)	28.9 (1.14)	28.6 (1.27)	0.251	28.7 (1.28)	28.9 (0.88)	0.342
Use of lipid-lowering agents		39 (38.2)	18 (38.3)	21 (38.2)	0.999	28 (33.7)	11 (57.9)	0.090
Use of tranquilizers/sedatives		23 (22.5)	6 (12.8)	17 (30.9)	**0.051**	17 (20.5)	6 (31.6)	0.362
Use of metformin		37 (36.3)	20 (42.6)	17 (30.9)	0.311	27 (32.5)	10 (52.6)	0.168
MedDiet adherence	*Mean (SD)*	7.36 (2.59)	6.79 (2.70)	7.85 (2.41)	**0.039**	7.42 (2.56)	7.11 (2.77)	0.652
Physical activity (METs x min/week)	*Mean (SD)*	2470 (2133)	2637 (2307)	2327 (1983)	0.472	2490 (2205)	2384 (1837)	0.829

*The chi-squared test for categorical variables and the Student’s t test for continuous variables were used to assess univariate differences between groups

The changes from baseline are presented as the means and 95%CI. Within-group changes over time were assessed using Cohen’s d effect size and linear mixed effects models. These models included participants as random effects, with time as the main explanatory variable and adjustments for sex and *APOE* covariates as appropriate. Gender and sex differences in cognitive changes relative to baseline were evaluated using analysis of covariance (ANCOVA) models. These models used changes from baseline to 1 year or 3 years (δ, representing time_2_-time_1_) as outcome variables, incorporating sex as an independent variable conditioned on the baseline score [57] in models for gender differences (i.e., unadjusted or ‘crude’ models), and adding sex covariates in models for sex differences. ANCOVA models adjusted for *APOE* covariates were also employed to examine *APOE-*related differences in changes across all study variables.

Generalized additive models (GAMs) with penalized regression splines and automatic smoothness estimation were used to examine the relationship between baseline eCBs concentrations and baseline cognitive performance, as well as to examine the relationship between 1- and 3-year changes in eCBs concentrations (δ) and the respective changes in cognition (δ). GAMs were chosen because they are not constrained by linear associations like generalized linear models [58]. The interaction effects between eCBs and sex or *APOE* genotype was tested. Accordingly, the smoothing parameter for eCBs was allowed to vary by sex or by *APOE* genotype (bivariable smoothing), so that different smoothing were generated for men and for women, as well as for *APOE-*ε4 carriers and noncarriers. GAMs were adjusted for sex or *APOE* covariates. The smoothness selection method chosen was restricted maximum likelihood (REML). The GAM fitting process was checked by examining the distribution of scaled residuals, marginally and plotted against fitted values, to ensure consistency with normality, constant variance as the mean increases, and a positive linear relationship between fitted and predicted values. A significant association was considered when the p-value of the smooth term of eCBs was below 0.05. In GAMs, the complexity of penalized smooths is measured by the effective degrees of freedom (EDF), which indicates the number of coefficients to be estimated in the model, minus any constraints. An EDF equal to 1 is equivalent to a linear relationship, and larger values indicate more wiggly terms [58]. To improve the interpretability of the results, when the relationship between eCBs and cognition estimated from GAMs was linear (EDF = 1), a linear regression model or ANCOVA model was then fitted to estimate the regression coefficient (β) and its 95%CI. However, when the relationship was nonlinear, smooth derivatives were estimated via finite differences [59] and an inflection point was determined when the derivative estimate was ≤ -0.05 or ≥ 0.05 (depending on the negative or positive trend of the curve). Next, a dummy variable indicating whether eCBs concentrations were below or above the inflection point was used to stratify participants. Finally, a linear model was fitted to estimate the regression coefficient of the linear part of the smooth curve.

The rates of missing data were greater for cognitive variables than for all the other variables, because the former were collected during the additional neuropsychological visit of the present substudy, whereas the latter were collected during the routine follow-up visits of the main PREDIMED-Plus study. All participants attended cardiovascular visits throughout the study; hence, missing data in such visits were assumed to be completely at random (MCAR). However, for the neuropsychological visits, attrition was present in 18 participants (17.7%) in the first year, and in 31 participants (30.4%) in the third year. Inverse probability weighting was used to address potential selection bias due to attrition in neuropsychological visits after 1 and 3 years. Inverse probability weights (IPWs) were calculated using a logistic regression model as the inverse probability of completing the follow-up based on observed related covariates. The area under the ROC curve was used for model selection, with values of 0.92 and 0.77 for IPWs at 1 and 3 years, respectively. Weight trimming was applied when necessary to avoid extreme weights, and weights were normalized to the sample size so that the sum of weights was equivalent to the total sample size. IPWs were included in all the analyses involving cognitive variables collected after 1 and 3 years.

All the analyses were performed with R statistical software, version 4.2.1. We used the R package ‘survey’ to compute the weighted analysis [60], the package ‘nlme’ to estimate linear mixed effects models [61], and the package ‘mgcv’ for GAMs [58].

## Results

### Description of the study population

The baseline characteristics of the study participants are included in Table 1. Briefly, 53.9% were women, 18.8% were *APOE*-ε4 carriers and the mean age was 65.6 ± 4.5 years. Most participants had obesity (84.3%) and nearly one-half had type 2 diabetes (46.1%). Participants scored 28.7 ± 1.2 points on the MMSE at baseline, so they performed within the normal range. Women had less years of education than men (10.2 ± 3.4 vs. 13.2 ± 4.4 years), were less active in the labor market (10.2% currently employed vs. 34.0% in men), and consumed more tranquilizers or sedatives (30.9% vs. 12.8%). Tobacco smoking was more common among men (17.0% vs. 3.6%), whereas er-MedDiet adherence was higher among women (7.8 ± 2.4 vs. 6.8 ± 2.7 points). The proportion of women was lower among *APOE*-ε4 carriers than among noncarriers (36.8% vs. 57.8%). Finally, the use of medication for lipid-lowering agents was greater in *APOE*-ε4 carriers than in noncarriers (57.9% vs. 33.7%). Compared to the overall PREDIMED-Plus cohort (*n* = 6,874), this subset of participants (*n* = 102) presented higher education level (Supplementary Table 1).

### Effect of gender and sex

#### Gender and sex differences in cognitive performance

**Fig. 1:**
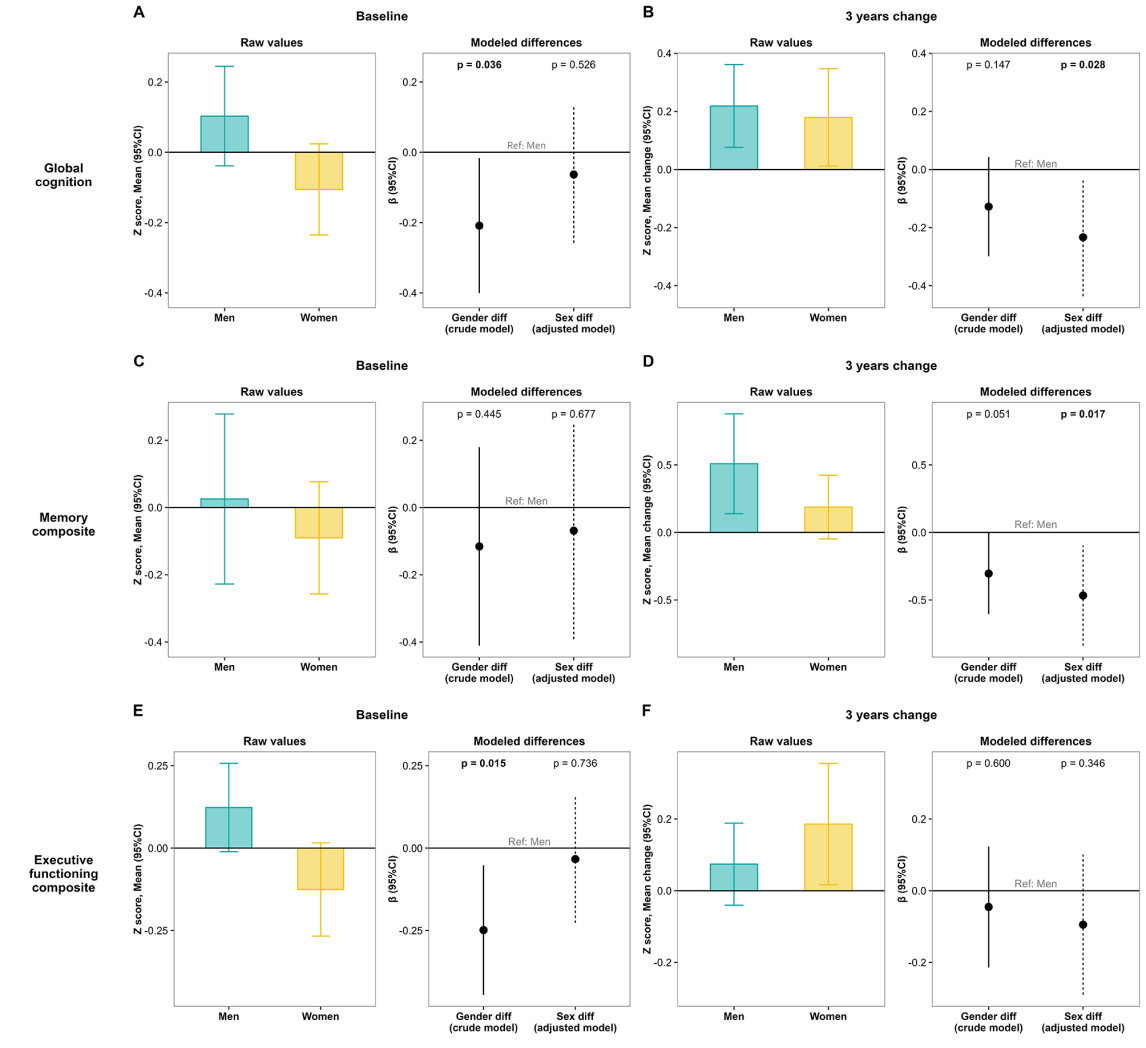
Sex and gender differences in baseline cognitive performance (**A, C, E**) and in cognitive change after 3 years of MedDiet intervention (**B, D, F**), represented in terms of global cognition (**A-B**), memory (**C-D**) and executive functioning (**E-F**) composites. Each plot consists of two panels: the left panel displays mean values (95%CI), while the right panel depicts modeled differences between men and women. A negative value of the modeled differences (β, 95%CI) indicates an effect favoring men. Gender differences are evaluated using unadjusted (crude) models, while sex differences are tested in models adjusted for gender-related factors (years of education, diabetes, use of tranquilizers or sedatives, use of lipid-lowering agents, baseline MedDiet adherence and baseline physical activity), age and *APOE* genotype. Bold values denote statistical significance at the *p* < 0.05 level. Further details are available in Supplementary Tables 1–2

At baseline, men exhibited higher performance in the global cognition (0.10 ± 0.50 vs. -0.11 ± 0.49 z score units) and executive function composites (0.12 ± 0.47 vs. -0.13 ± 0.53 z score units), partially attributed to differences in processing speed and decision-making abilities (Supplementary Table 2). Moreover, men exhibited greater visual memory performance, while women outperformed men in long-term verbal memory. The effect size of gender differences in baseline cognitive performance was moderate to high (Cohen’s d ranging from 0.42 to 0.76). The proportion of variance in cognition explained by gender was 3.4% for the global cognition composite (*p* = 0.036), 4.8% for the executive functioning composite (*p* = 0.015), 6.2% for decision-making abilities (*p* = 0.008), 9.7% for processing speed (*p* < 0.001), 10% for long-term visual memory (*p* < 0.001), and 11.9% for short-term visual memory (*p* < 0.001). However, these differences vanished in multivariable-adjusted models of sex differences (Fig. 1A, C, E), with men exhibiting higher performance than women solely in visual memory.

#### Gender and sex differences in cognitive change

After 1 year, there were no significant gender or sex differences in cognitive change as assessed by cognitive composites. Both genders demonstrated small improvements in global cognition (Cohen’s d of 0.32 (*p* < 0.001) in men, and 0.26 (*p* = 0.048) in women), primarily due to improvements in memory (Cohen’s d of 0.42 (*p* < 0.001) in men and 0.40 (*p* = 0.018) in women) (Supplementary Table 3). However, in specific domains such as visual memory or inhibition, men showed greater improvements than women after 1 year, with Cohen’s d values of gender differences ranging from 0.35 to 0.51.

After 3 years, gender and sex differences in memory change were observed, favoring men (Fig. 1B, D, F). Specifically, the Cohen’s d effect size of memory changes in men was 0.40 while that in women was 0.25, leading to significant gender (β = -0.30, 95%CI -0.61 to 0.00; *p* = 0.051) and sex differences (β = -0.47, 95%CI -0.84 to -0.10; *p* = 0.017) in terms of memory change. After 3 years there were also significant sex differences in global cognitive changes favoring men (β=-0.23, 95%CI -0.44 to -0.03; *p* = 0.028), as well as in inhibition and attention measured with the Stroop interference test.

#### Relationship between eCBs and cognition by sex

**Fig. 2:**
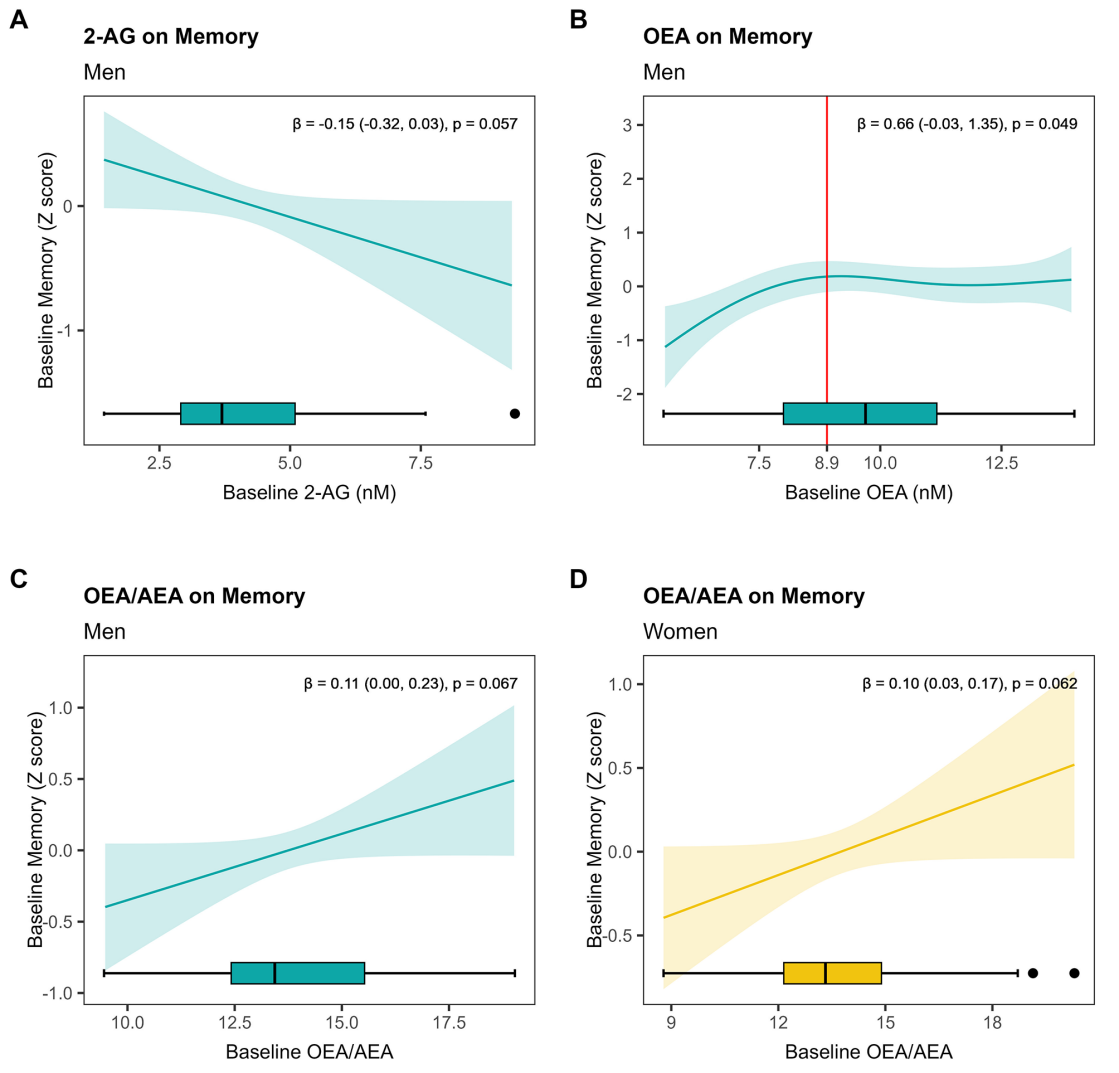
Estimated smoothness of baseline eCBs or NAEs on cognitive performance by sex derived from GAMs. The Y-axis depicts the partial effect of baseline 2-AG on baseline global cognition in men (**A**), baseline OEA on baseline memory in men (**B**), baseline ratio OEA/AEA on baseline memory in men (**C**), and baseline ratio OEA/AEA on baseline memory in women (**D**). The shaded area is the standard-error confidence intervals. Red lines indicate the inflection point for non-linear relationships. Regression coefficients (β) and 95%CI were obtained from linear models for improving the interpretability of linear relationships. The p-value indicates the significance of the smooth term in the GAM

At baseline, 2-AG was negatively and linearly associated with memory performance in men (β = -0.15, 95%CI -0.32 to 0.03, p_GAM_=0.057) (Fig. 2A). A nonlinear relationship was also found between OEA concentrations and memory in men (Fig. 2B). Accordingly, an increase in OEA was positively associated with memory performance until it reached a concentration ≥ 8.9 nM (β = 0.66, 95%CI -0.03 to 1.35; p_GAM _= 0.049); from there on, there was no relationship between OEA and memory. In turn, the OEA/AEA ratio was positively and linearly associated with memory performance in both men (β = 0.11, 95%CI 0.00 to 0.23; p_GAM _= 0.067) and women (β = 0.10, 95%CI 0.03 to 0.17; p_GAM _= 0.062) (Fig. 2C, D).

**Fig. 3:**
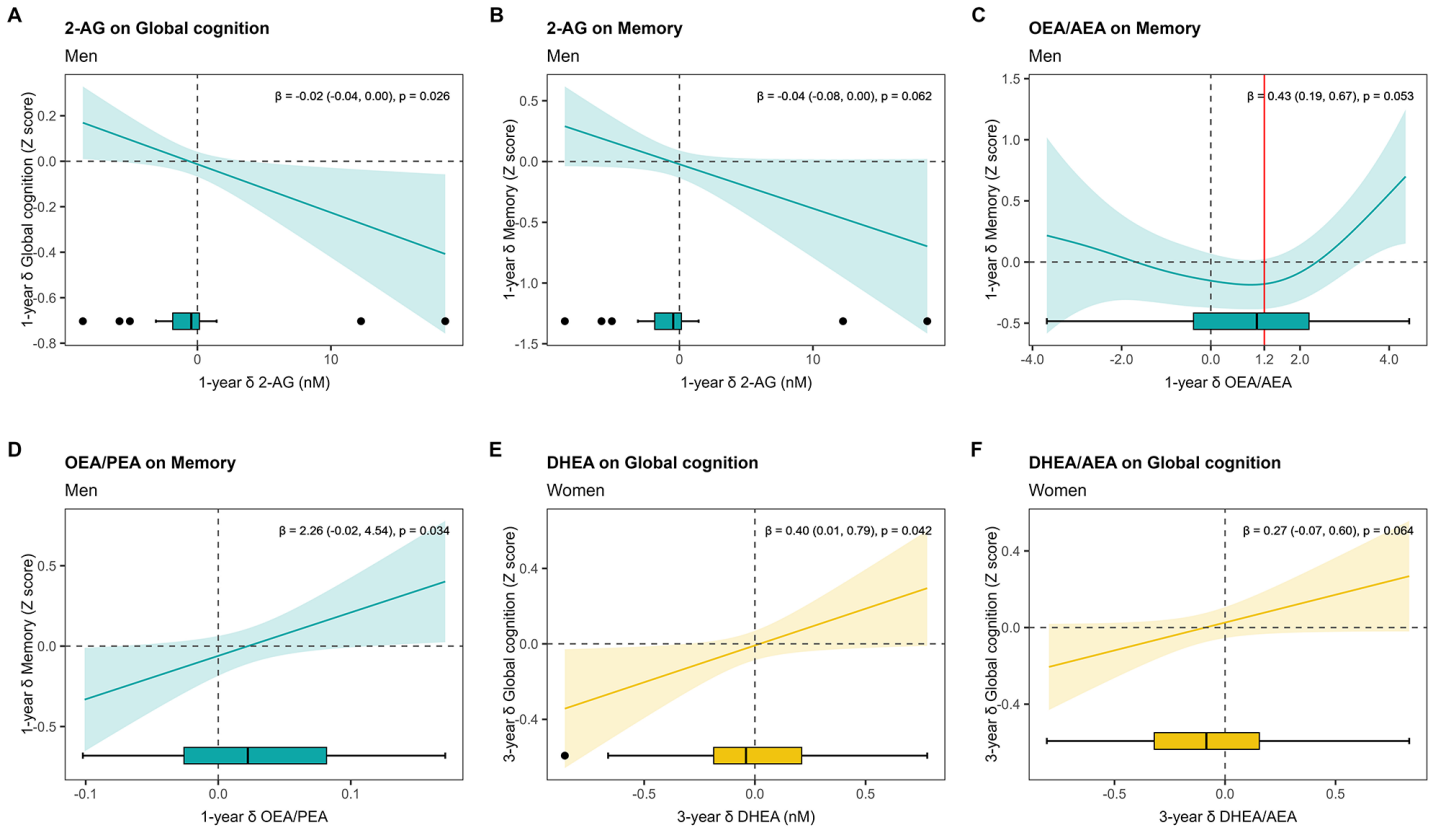
Estimated smoothness of change in eCBs or NAEs on cognitive change by sex derived from GAMs. The Y-axis depicts the partial effect of 1-year change in 2-AG on 1-year change in global cognition in men (**A**), 1-year change in 2-AG on 1-year change in memory in men (**B**), 1-year change in the ratio OEA/AEA on 1-year change in memory in men (**C**), 1-year change in the ratio OEA/PEA on 1-year change in memory in men (**D**), 3-year change in DHEA on 3-year change in global cognition in women (**E)**, and 3-year change in the ratio DHEA/AEA on 3-year change in global cognition in women (**F**). The shaded area is the standard-error confidence intervals, and δ indicates change after 1 or 3 years. Red lines indicate the inflection point for non-linear relationships. Regression coefficients (β) and 95%CI were obtained from ANCOVA models for improving the interpretability of linear relationships. The p-value indicates the significance of the smooth term in the GAM

After 1 year, within-subject changes in 2-AG were negatively associated with changes in global cognition (β = -0.02, 95%CI -0.04 to 0.00; p_GAM _= 0.026) and memory performance (β = -0.04, 95%CI -0.08 to 0.00; p_GAM _= 0.062) in men, and these relationships were linear (Fig. 3A, B). Along with the baseline results, there was also a nonlinear association between changes in the OEA/AEA ratio and memory changes in men (Fig. 3C), showing that an increase in this ratio, but not a decrease, was positively associated with memory change (inflection point at δ ≥ 1.2; β = 0.43, 95%CI 0.19 to 0.67; p_GAM _= 0.053). After 1 year, increases in the OEA/PEA ratio were linearly associated with memory improvements in men (β = 2.26, 95%CI -0.02 to 4.54; p_GAM _= 0.034) (Fig. 3D).

After 3 years, increases in DHEA concentrations in women were linearly associated with improvements in global cognition (β = 0.40, 95%CI 0.01 to 0.79; p_GAM _= 0.042) (Fig. 3E). Similarly, a linear relationship was found between 3-year changes in the DHEA/AEA ratio and global cognitive changes in women (β = 0.27, 95%CI -0.07 to 0.60; p_GAM _= 0.064) (Fig. 3F).

### Effect of *APOE* genotype

#### *APOE* differences in cognition

At baseline, there were no differences in cognitive performance based on *APOE* genotype (Supplementary Table 4). After 1 year of MedDiet intervention, both *APOE*-ε4 carriers and noncarriers exhibited improvements in global cognition and memory (*p* < 0.05), with no significant differences between groups (Supplementary Table 5). However, the Cohen’s d effect size of differences ranged 0.30 to 0.57, favoring *APOE*-ε4 carriers. After 3 years, global cognition and executive functioning composites improved in *APOE*-ε4 noncarriers but, on average, there was no significant change in these composites in *APOE*-ε4 carriers. However, although the Cohen’s d effect size of differences in cognitive change was moderate (-0.52 for global cognition and − 0.46 for memory), multivariable-adjusted models showed no significant differences between groups, except for the specific domain of visuoconstructive praxis and attention favoring *APOE*-ε4 noncarriers (Cohen’s d of -0.74, *p* = 0.010).

#### *APOE* differences in eCBs and NAEs

At baseline, the concentrations of eCBs and NAEs did not differ according to *APOE* genotype (Supplementary Table 6). After 6 months of MedDiet intervention, 2-AG, AEA, and several NAEs (OEA, PEA, DHEA, DGLEA, LEA, POEA, and SEA) decreased in *APOE*-ε4 noncarriers but remained unchanged in *APOE*-ε4 carriers (Supplementary Table 7). Larger differences between groups were observed for OEA (Cohen’s d = 1.08, *p* = 0.009) and PEA (Cohen’s d = 1.09, *p* = 0.009), and smaller differences were detected for AEA (Cohen’s d = 0.34, *p* = 0.003), LEA (Cohen’s d = 0.38, *p* = 0.007) and DEA (Cohen’s d = 0.14, *p* = 0.006) and DGLEA (Cohen’s d = 0.10, *p* = 0.048). After 1 year, *APOE*-ε4 noncarriers exhibited greater increases in the PEA/AEA ratio (Cohen’s d=-1.02, *p* = 0.031). Similarly, *APOE*-ε4 noncarriers showed greater increases in the DHEA/AEA ratio after 1 year (Cohen’s d=-0.36, *p* = 0.083) and 3 years (Cohen’s d=-0.30, *p* = 0.015).

#### *APOE* differences in cardiovascular and lifestyle risk factors

At baseline, cardiovascular and lifestyle risk factors did not differ according to *APOE* genotype (Supplementary Table 8). Between-group differences in changes in these factors were detected in terms of diastolic blood pressure and total cholesterol (Supplementary Table 9). Accordingly, after 6 months, *APOE*-ε4 carriers showed greater reductions in diastolic blood pressure than noncarriers (mean change of -8.0 vs. -3.6 mmHg, Cohen’s d= -1.64, *p* = 0.053). Similarly, after 1 year, *APOE*-ε4 carriers experienced greater reductions in total cholesterol than noncarriers (mean change of -9.0 vs. 3.7 mg/dL, Cohen’s d= -2.09, *p* = 0.045).

#### Association between eCBs and cognition by *APOE* genotype

**Fig. 4:**
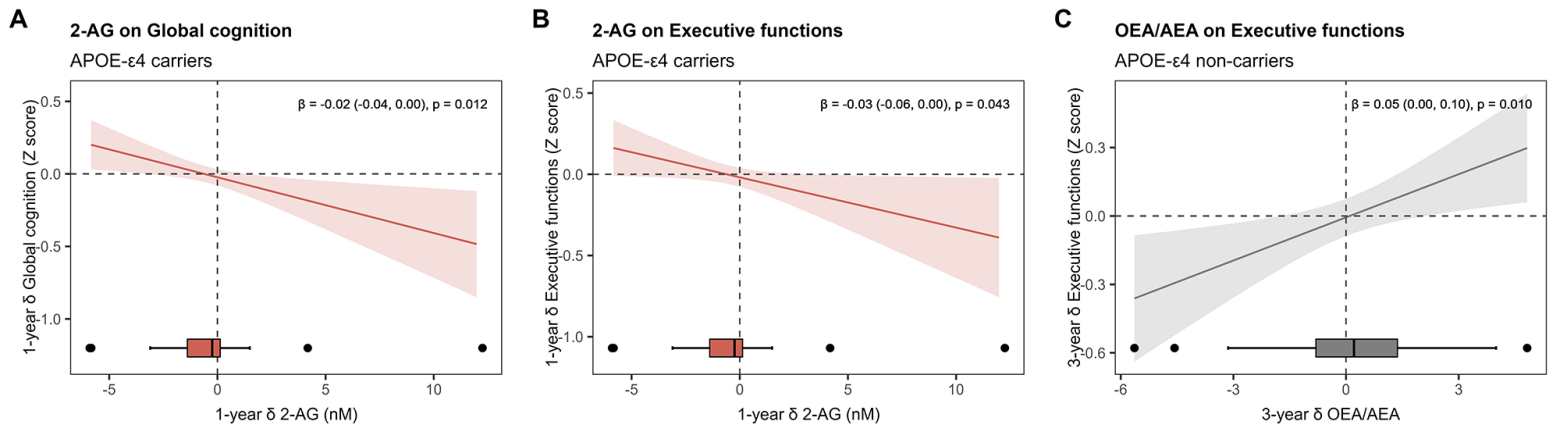
Estimated smoothness of change in eCBs or NAEs on cognitive change by *APOE*-ɛ4 genotype derived from GAMs. The Y-axis depicts the partial effect of 1-year change in 2-AG on 1-year change in global cognition in *APOE*-ɛ4 (**A**), 1-year change in 2-AG on 1-year change in executive functions in *APOE*-ɛ4 carriers (**B**), and 3-year change in the ratio OEA/AEA on 3-year change in executive functions in *APOE*-ɛ4 noncarriers. The shaded area is the standard-error confidence intervals, and δ indicates change after 1 or 3 years. Regression coefficients (β) and 95%CI were obtained from ANCOVA models for improving the interpretability of linear relationships. The p-value indicates the significance of the smooth term in the GAM.

As shown in Fig. 4A-B, within-subject changes in 2-AG concentrations after 1 year were negatively associated with changes in global cognition (β = -0.02, 95%CI -0.04, 0.00; p_GAM _= 0.012) and executive functions (β = -0.03, 95%CI -0.06, 0.00; p_GAM _=0.043) among *APOE*-ε4 carriers. In turn, within-subject change in the OEA/AEA ratio after 3 years was positively associated with change in executive function among *APOE*-ε4 noncarriers (Fig. 4C), and this relationship was linear (β = 0.05, 95%CI 0.00, 0.10; p_GAM _= 0.010).

## Discussion

### Main findings

In this prospective study, we examined the interplay between gender, sex, cognitive performance, and the modulation of eCBs in older adults with metabolic syndrome and overweight or obesity participating in a MedDiet intervention for three years. We also explored the influence of the *APOE*-ε4 genotype on the cognitive and metabolic responses to MedDiet intervention, as well as its role in modulating the relationship between eCBs and cognitive changes. At baseline, men exhibited superior performance in executive functioning and global cognition than women. This disparity was primarily attributed to gender-related health inequalities rather than to inherent biological sex differences. Over the course of the three-year MedDiet intervention, both genders experienced improvements in memory and global cognition. However, improvements were more pronounced in men after considering gender-related factors, suggesting biological sex differences in the cognitive response to a MedDiet intervention. Moreover, 2-AG concentrations were negatively associated with cognitive performance and 1-year cognitive changes in men and *APOE*-ε4 carriers. A higher OEA/AEA ratio also indicated better memory performance in both genders, and an increase in this ratio after 1 year was found to be associated with memory improvements in men and *APOE*-ε4 noncarriers. Finally, changes in DHEA or the DHEA/AEA ratio were positively associated with 3-year cognitive changes in women. To our knowledge, this is the first study in examining the association between eCBs and cognitive change in humans.

### Gender and sex differences in cognitive change

Although female gender is a well-known risk factor for dementia [62], few studies to date have examined gender and sex differences in response to interventions aiming to forestall cognitive decline [63], particularly the MedDiet [23, 64]. Consistent with the findings of previous studies [65], at baseline men performed better than women in short- and long-term visual memory, decision-making ability and processing speed, whereas women performed better in long-term verbal memory. In a previous cohort study that followed 34,349 participants for ~ 8 years, women also had faster rates of decline in global cognition than men [66]. A reduction in estradiol and estrone levels during menopause could exacerbate the effects of cognitive aging [65]. Accordingly, a recent study of surgically menopausal women showed impaired verbal memory and working memory performance, but working memory was maintained among those who received estradiol therapy [67].

In a previous study with a larger sample of PREDIMED-Plus participants, we examined the sex-specific effect of the MedDiet on global cognition, memory and executive function composites, but not on specific cognitive domains [23]. Consistent with our previous report, in this study men experienced greater cognitive improvements than women in global cognition and memory as well as in the specific domains of long-term verbal memory, inhibition and attention. The differential effect of the MedDiet on cardiovascular risk factors may partly explain the greater cognitive benefits observed in men [2, 23]. Accordingly, men also presented greater glycemic and cardiovascular benefits after the MedDiet intervention, including greater reductions in body weight, triglycerides and insulin resistance [21], despite the lack of sex differences in MedDiet adherence or food categories consumed. Moreover, the presence of metabolic syndrome poses a greater risk of cognitive decline for postmenopausal women than for men of the same age due to the differential distributions of central adiposity, lipid profiles and hormones [68, 69].

Thus far, three large long-term multidomain lifestyle RCTs have been conducted among cognitively unimpaired older adults: the FINGER study (Finnish) [70], the PreDIVA study (Dutch) [71] and the MAPT Study (French) [72]. In the FINGER Study, the overall beneficial effects of the 2-year lifestyle intervention did not vary by sex [73, 74]. In the PreDIVA study, a 6-year intervention failed to influence dementia incidence and cognitive function, but there were no sex differences [71]. In a pooled analysis of the PreDIVA and MAPT trials, the interaction between the intervention and sex was not significant [75]. However, dementia risk reduction, evaluated with the CAIDE and FINRISK risk scores was greater in women than in men [76], which contrasts with our findings that men experience greater cardiovascular benefits and weight reductions than women [21]. Several other large lifestyle trials, such as the HATICE study (multinational) [77], the Diabetes Prevention Program Outcome Studies (USA) [78], and the Look AHEAD study (USA) [79, 80], also reported no sex differences in cognitive outcomes.

Cognitive reserve could also contribute to the observed sex differences in cognitive change [81], as sex and gender interact in a process called ‘embodiment’ [82]. Cognitive reserve depends on education, occupational complexity, and cognitive activity, factors that are more related to the social construct of gender than to biological sex [83]. On average, men in our cohort had 3 more years of education than women. Furthermore, our cohort of participants was born between 1940 and 1961. Women at that time had not only limited access to education but also, above all, limited access to the labor market. These results are consistent with previous studies that showed that cognitive reserve is an important mediator of the association between lifestyle factors and cognition [84]. However, we cannot discard other potential factors that could interact with the MedDiet to affect cognition in a sex-specific manner such as genetic, lifestyle and psychological factors, or sex-specific vulnerability to AD pathology [51, 63, 66, 68, 85].

### *APOE* differences in cognitive change

During the first year of MedDiet intervention there were no significant differences in cognitive changes according to *APOE* genotype. However, the effect size of positive cognitive changes was generally greater among *APOE*-ε4 carriers than among noncarriers, which is in accordance with the two-year follow-up of the FINGER trial [24]. In contrast, after 3 years, the effect size of cognitive changes was greater among *APOE-ε4* noncarriers, even though the results were only statistically significant for visuoconstructive praxis and attention. Longitudinal studies examining the effect of *APOE* status on cognitive change have reported mixed results [86, 87], with studies pointing to greater declines in episodic memory, executive functions, processing speed and visuospatial ability and other studies reporting no differences in cognitive change [87]. From the first year onwards, the intensity of the PREDIMED-Plus intervention decreased in terms of the number of follow-up visits [32–34], as the goal was to sustain the 6-month and 1-year cardiometabolic and weight changes in the long run. The reduction in intervention intensity may explain why greater cognitive benefits were observed among *APOE*-ε4 noncarriers after 3 years of follow-up. This might be further supported by the absence of differences in intervention adherence and cardiometabolic risk factors according to *APOE* genotype.

There is evidence suggesting that women carriers of the *APOE*-ε4 allele have an increased risk of developing AD earlier than men carriers [88, 89]. However, even though the *APOE*-ε4 subgroup had a greater proportion of men, the effect size of *APOE* differences in cognitive change after 3 years was slightly larger than that of sex differences. This phenomenon should be explored in future studies stratified by both sex and *APOE* genotype.

### *APOE* differences in eCBs and NAEs

*APOE* differences in the modulation of AEA and other NAEs (OEA, PEA, DHEA, DGLEA, LEA, POEA, and SEA) after MedDiet intervention may result from alterations in lipid signaling that have already been described among *APOE*-ε4 carriers [22, 90]. Impairments in lipid transport machinery in the presence of the APOE4 isoform involve the neural receptor sortilin and the fatty acid binding protein 7 (FABP7), and have been shown to ultimately disrupt proper intracellular lipid handling and action [22, 90, 91].

### Association between 2-AG and cognitive changes

2-AG plasma concentrations decreased after 6 months of exposure to a MedDiet intervention, and remained lower during the three years of follow-up [21]. This decrease in 2-AG after 1 year was associated with improved global cognition in men and *APOE*-ε4 carriers. Although AEA also binds to the cannabinoid receptor type 1 (CB_1_R), it was not associated with cognitive changes. Moreover, AEA decreased after 6 months of MedDiet intervention but rose to baseline concentrations after 1 year [21]. Our results are in agreement with previous studies showing that 2-AG, but not AEA, is dynamically coupled to hippocampal neural activity with high spatiotemporal specificity, supporting that 2-AG is the dominant activity-dependent eCB in the hippocampus [92]. Notably, brain 2-AG concentrations are ~ 170 times higher than those of AEA [93].

Our results are consistent with studies showing that 2-AG concentrations are elevated in the plasma samples of AD patients [17, 94] and in the brain samples of AD mouse models [95], and that elevated 2-AG may aggravate synapse impairment in AD [96] and obese mouse models [97, 98]. Cross-sectional studies also support the inverse correlation between 2-AG concentrations and cognitive performance, in cognitively normal individuals [15, 16] or those with AD [17].

The relationship between 2-AG and cognition was limited to men and *APOE*-ε4 carriers, even though we did not find sex- or *APOE*-differences in the modulation of 2-AG by the MedDiet [21]. In our population, peripheral 2-AG strongly correlated with triglycerides in men but not in women [21]. Triglycerides have been shown to cross the blood-brain-barrier and induce central insulin resistance [99]. Therefore, the reduction in 2-AG observed in men after a MedDiet intervention could be a marker of metabolic improvements (e.g. reductions in triglycerides), and could indicate a reduction in insulin resistance, which is a known risk factor for cognitive decline in individuals with metabolic syndrome [100]. This hypothesis would be supported by previous findings in the same population showing that weight reductions after a MedDiet intervention were associated with cognitive benefits in men but not women [23]. This interpretation also aligns with previous reports showing that adverse effects of vascular and metabolic risk factors increase the risk of cognitive decline in men and women through partly different mechanisms [85, 101, 102]. However, future studies should confirm the observed sex and *APOE* differences observed in the relationship between 2-AG and cognitive changes and should also explore the specific mechanisms underlying these differences.

### Associations between DHEA and DHEA/AEA ratio and cognitive changes

Within-subject changes in DHEA concentrations or the DHEA/AEA ratio after 3 years of MedDiet intervention were positively associated with changes in global cognition in women. These findings support the hypothesis that DHEA mediates the effects of DHA on cognition [103–106]. DHEA is also known as ‘synaptamide’ because it promotes neurogenic differentiation [107] and enhances synaptogenesis, neuritogenesis, and glutamatergic synaptic activity [103, 108]. In vitro experiments have shown that DHEA also protects against neuroinflammation [109], which is important given that dysregulated inflammation is a common feature of several neurodegenerative diseases, including AD [110]. The therapeutic potential of DHEA has also been observed in rodent studies, showing protection against neuroinflammation and, more importantly, cognitive impairment [111, 112]. Women displayed higher DHEA concentrations, although no sex differences in DHA were detected [21]. Moreover, baseline DHEA and DHA concentrations correlated in women, but not in men [21]. Although sex differences in omega-3 fatty acid metabolism have been reported [113], studies addressing sex differences in the association between omega-3 supplementation and cognitive changes are lacking [27].

### Association between OEA and cognitive performance

At baseline, a logarithmic relationship was observed between OEA and memory; hence, an increase in OEA was associated with improved memory performance until certain concentrations were reached; thereafter, no relationship was observed despite increasing concentrations. This relationship was specific to men, who also had lower OEA concentrations than women [21]. OEA is involved in peripheral appetite regulation, as oral administration of OEA decreases food intake and increases satiety [114–116]. In line with our findings, OEA administration to rats facilitated memory consolidation [117]. It has been hypothesized that OEA produced in the gut after consuming a fat-rich meal initiates an integrated response via vagal afferents, reaching satiety centers to control feeding behavior, which may coincide temporally with memory consolidation of salient information about the spatial and emotional context in which the meal was consumed [118]. Moreover, in diabetic mice, OEA administration has been shown to lower hyperglycemia and recover cognitive performance, reduce dementia markers and inhibit hippocampal neuron loss and neuroplasticity impairments [119]. OEA also modulates cognitive deficits induced by MDMA (3,4-methylenedioxymethamphetamine) in mice [120], and induces recovery of cognitive deficits due to a cerebral ischemic insult in rats [121]. In an RCT of patients with acute ischemic stroke, OEA supplementation improved inflammation, oxidative stress, and lipid and biochemical parameters [122].

### Association between OEA/AEA or OEA/PEA ratios and cognitive changes

The use of NAEs ratios rather than the concentrations of individual compounds has been recently proposed to improve the understanding of the regulation of the endocannabinoid system [20, 123]. In both men and women, we observed that the baseline OEA/AEA ratio was positively and linearly associated with memory. In men, OEA/AEA was positively correlated with MedDiet adherence, and negatively correlated with BMI and insulin resistance (HOMA-IR) [21]. In women, baseline OEA/AEA negatively correlated with HbA1c and fasting plasma glucose, but not with MedDiet adherence or HOMA-IR [21]. Insulin resistance, hyperglycemia and obesity are known risk factors for AD, which supports the positive effects of the OEA/AEA ratio on memory performance in a sex-specific manner.

After 1 year, changes in OEA/AEA ratio were associated with cognitive changes in men and *APOE*-ε4 noncarriers. This relationship was nonlinear, particularly in men; hence an increase in this ratio, but not a reduction, was associated with cognitive benefits. Similar nonlinear associations have been identified between fatty acids and the incidence of type 2 diabetes [124] or between healthy Nordic foods and all-cause mortality [125]. In the field of AD, non-linear relationships have also been observed between β-amyloid and tau biomarkers and cognitive change [126]. The European Medicines Agency (EMA) and the US Food and Drug Administration (FDA) have also discussed nonlinear models as clinical trial design tools for studying AD [127]. Overall, these findings support complex nonlinear relationships between eCBs and cognition, which could explain the high level of inconsistency in previous studies, as linear associations have traditionally been assumed. However, establishing optimal doses or changes in eCBs to have an impact on cognition is not straightforward, as analyses of isolated eCBs do not consider synergistic or antagonistic effects, which is recognized as an ‘entourage effect’ in the field of eCBs [128]. Moreover, this is the first study to show that the effects of NAEs balance on cognition may differ according to sex and *APOE* genotype, which adds complexity to the understanding of the role of the endocannabinoid system in cognition.

Ultimately, a positive linear relationship was found between the 1-year changes in the OEA/PEA ratio and 1-year memory changes in men. Specifically in men, increases in the OEA/PEA ratio were also associated with the achievement of clinically meaningful weight reductions of more than 8% of body weight and with reductions in insulin resistance [21]. These results support the use of the OEA/PEA ratio as a marker of metabolic and cognitive improvements in men. The mechanism underlying the effects of OEA/AEA and OEA/PEA ratios on cognition could be related to their molecular targets. OEA and PEA activate both peroxisome proliferator-activated receptor α (PPAR-α) and transient receptor potential cation channel subfamily V member 1 (TRPV1). TRPV1 is also a target of AEA and 2-AG [129]. Thus, PPAR-α could be responsible for a possible association with cognitive amelioration, whereas TRPV1 could counteract this potential effect at higher concentrations [130].

### eCBs as biomarkers of cognitive benefits of the MedDiet

**Fig. 5:**
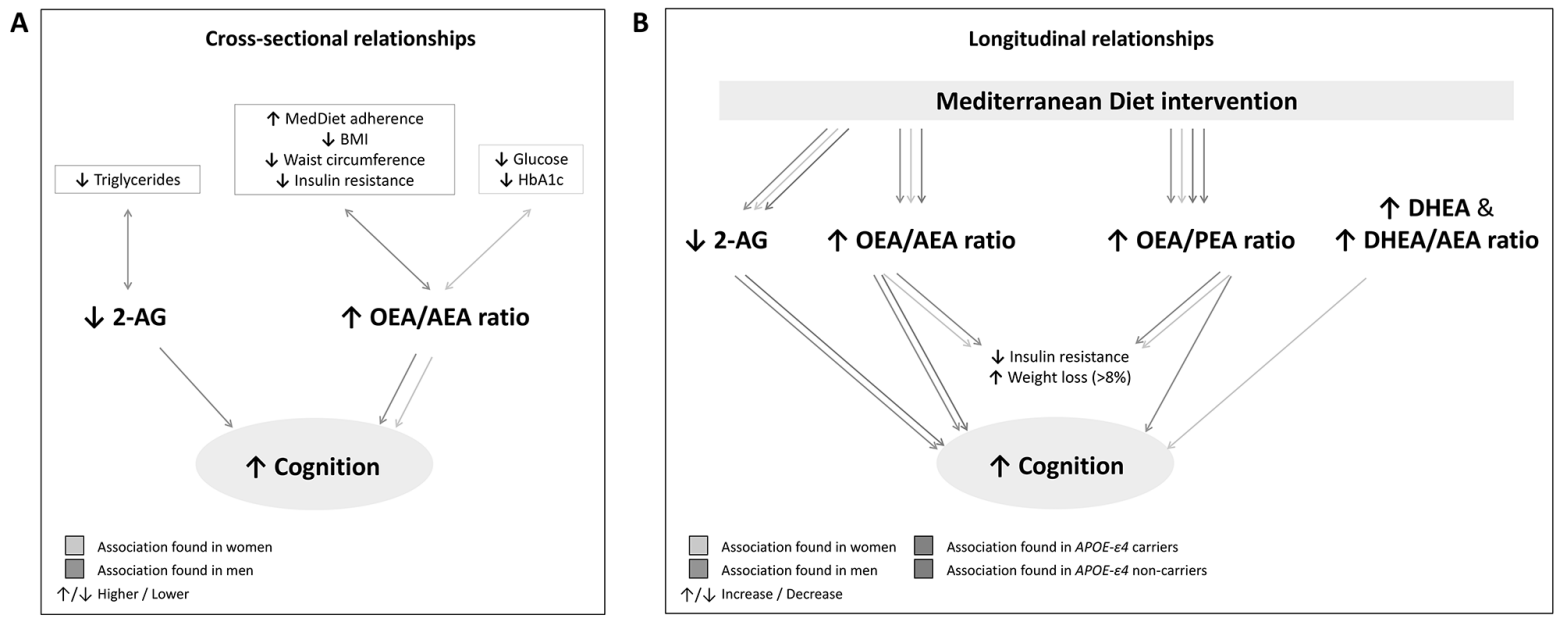
Proposed mechanism of cognitive change by the MedDiet via the endocannabinoid system

In summary, the findings of this study, together with an earlier study conducted in the same cohort [21], shed light on a potential mechanism through which the MedDiet may benefit cognition: modulation of the endocannabinoid system (Fig. 5). The OEA/AEA ratio emerges as a key biomarker related to the cognitive and metabolic benefits of the MedDiet in both sexes. This ratio may indicate improvements in glucose homeostasis, resulting in cognitive improvements. It involves the interaction between two receptors with potential opposing effects: the PPAR-α and the CB_1_R, amenable to modulation with pharmacological treatments. Furthermore, our findings suggest that reducing or controlling 2-AG concentrations could be important for men and *APOE*-ε4 carriers to prevent cognitive decline. Ultimately, we identified two potential sex-specific biomarkers of MedDiet-derived cognitive benefits: the OEA/PEA ratio in men and DHEA in women.

### Limitations

The main limitation of this study is the small sample size of *APOE*-ε4 carriers (*N* = 19, 18.8%), although this was expected given the overall sample size (*N* = 102) [131]. The limited sample size, coupled with the exploratory nature of the present study, could impact the robustness of the study findings. Thus, our results need to be replicated in larger cohorts of participants, particularly among *APOE*-ε4 carriers. Moreover, even though risk estimates for *APOE*-ε4 carriers are usually greater for women than for men [88, 89], we could not examine the interaction between sex and *APOE* genotype due to the limited number of *APOE-ε4* carriers. However, all the analyses of sex differences were adjusted for *APOE* genotype and vice versa. Another limitation is that the inflection points (or ‘change points’) detected in the nonlinear models (GAMs) were not validated in an external cohort, as they were only used for the sake of interpretability. Moreover, the studied population was restricted to older adults with metabolic syndrome who had overweight or obesity, which could affect the generalizability of our findings. Finally, there were losses in the evaluation of the cognitive function after 1 year (17.7%) and 3 years (30.4%). They were not unexpected given the burden of such visits and the fact that the neuropsychological visits were performed on different days than the routine visits, when plasma samples for eCBs were collected. To address this missing data problem, all the analyses of 1-year and 3-year changes in each cognitive test were computed using inverse probability weighting. Weights were applied to the subjects with no missing outcome data, so it was assumed that those who were unsuccessfully followed presented cognitive scores that could be accurately estimated from those successfully followed.

## Conclusions

In this study, we observed that a high adherence to the MedDiet not only contributed to preventing cognitive decline but also provided a global benefit to individuals’ cognition. However, the effect size of cognitive improvements was small. Prevention of the decline in cognitive performance typical of aging over a three year period is likely the most relevant effect.

Sex was identified as a determinant of cognitive change. The study of sex differences in the field of AD is increasingly recognized as a key priority in research and clinical development [132]. Our results support the idea that gender inequalities over the life course, together with biological sex differences, contribute to the success of lifestyle interventions. Understanding which individuals are most likely to benefit from lifestyle interventions has direct implications for the design of future studies and interventions. This knowledge underscores the need for personalized preventive strategies.

Despite significant advances in the clinical and biological understanding of AD, the unsatisfactory results of pharmacological RCTs have highlighted the limited knowledge about the factors and pathways driving cognitive changes. In this study, we examined pathways related to the lipid homeostasis, including endocannabinoids (eCBs) and eCB-like compounds (NAEs), as potential mechanisms underlying cognitive changes. Furthermore, we evaluated how sex and *APOE* genotype modify the relationship between eCBs and cognition. Although the interaction between sex and diet is complex, developing a therapeutic approach that modulates the endocannabinoid system (e.g., partially inhibiting 2-AG with peripheral or neutral CB_1_R antagonists or allosteric modulators of CB_1_R) and improves the activity of the PPARα receptor (e.g., with synthetic PPAR-α agonists) may well be of interest in the context of improving cognitive performance. The results support the idea that nutritional interventions and pharmacological treatment could represent a combined approach for preventing cognitive decline [133].

### Electronic supplementary material

Below is the link to the electronic supplementary material.

Supplementary Material 1

## Data Availability

The datasets presented in this article are not readily available because there are restrictions on the availability of data for the PREDIMED-Plus trial, due to the signed consent agreements around data sharing. The researchers wishing to access the PREDIMED-Plus dataset generated and/or analyzed during the current study can make a request to the PREDIMED-Plus trial Steering Committee chair. The requests to access the datasets should be directed to JS-S, jordi.salas@urv.cat.

## Abbreviations

2-AG: 2-arachidonoylglycerol
AEA: anandamide or *N-*arachidonoyl-ethanolamine
AD: Alzheimer’s disease
APOE: Apolipoprotein E
BMI: body mass index
CB_1_R: cannabinoid receptor type 1
DEA: *N-*docosatetraenoylethanolamine
DGLEA: *N-*dihomo-ɣ-linolenoyl ethanolamide
DHA: docosahexaenoic acid
DHEA: *N-*docosahexaenoylethanolamine
eCBs: endocannabinoids
er-MedDiet: energy-reduced Mediterranean diet
GAMs: generalized additive models
HbA1c: glycosylated hemoglobin
HDL-c: high-density lipoprotein cholesterol
HOMA-IR: homeostasis model assessment of insulin resistance
LDL-c: low-density lipoprotein cholesterol
LEA: *N-*linoleoylethanolamine
MedDiet: Mediterranean diet
NAEs: *N-*acylethanolamines
OEA: oleoylethanolamide
PEA: palmitoylethanolamide
POEA: *N-*palmitoleoylethanolamine
RCT: randomized-controlled trial
SEA: *N-*stearoylethanolamine
