# Supplementary material for: Relationship between sex, APOE genotype, endocannabinoids and cognitive change in older adults with metabolic syndrome during a 3-year Mediterranean diet intervention
Source: Nutr J. 2024 Jun 12;23:61. doi: 10.1186/s12937-024-00966-w (PMC11167771; doi:10.1186/s12937-024-00966-w)
Supplement: Supplementary file 1 — Supplementary Material 1 [file 12937_2024_966_MOESM1_ESM.docx]

**SUPPLEMENTARY MATERIAL**

**Index**

[Supplementary Tables 2](#_Toc164239252)

[Supplementary Table 1. Comparison of the sub-cohort analyzed in the present with the overall PREDIMED-plus cohort and the PREDIMED-plus-Cognition cohort 2](#_Toc164239253)

[Supplementary Table 2. Sex differences in baseline cognitive performance. 3](#_Toc164239254)

[Supplementary Table 3. Sex differences in cognitive change after 1 and 3 years. 4](#_Toc164239255)

[Supplementary Table 4. Differences in baseline cognitive performance by *APOE*-ɛ4 genotype 5](#_Toc164239256)

[Supplementary Table 5. *APOE*-ɛ4 differences in cognitive change after 1 and 3 years 6](#_Toc164239257)

[Supplementary Table 6. Differences in baseline concentrations of eCBs and NAEs by *APOE*-ɛ4 genotype 7](#_Toc164239258)

[Supplementary Table 7. Changes in eCBs and NAEs concentrations by APOE-ɛ4 genotype 8](#_Toc164239259)

[Supplementary Table 8. Differences in baseline cardiovascular and lifestyle risk factors by *APOE*-ɛ4 genotype 9](#_Toc164239260)

[Supplementary Table 9. Changes in cardiovascular and lifestyle risk factors by *APOE*-ɛ4 genotype 10](#_Toc164239261)

# Supplementary Tables

| Supplementary Table 1. Comparison of the sub-cohort analyzed in the present with the overall PREDIMED-plus cohort and the PREDIMED-plus-Cognition cohort | | | |
| --- | --- | --- | --- |
| **Baseline characteristics** | **PREDIMED-Plus Cohort^1^**  **(N=6,874)** | **PREDIMED-Plus-*Cognition* Cohort**  **(N=487)** | **PREDIMED-Plus-*Cognition*,**  **recruited at HMRI (Barcelona) (N=102)** |
| Age (years; mean (SD) | 65.0 (4.9) | 65.2 (4.7) | 65.6 (4.5) |
| Female sex (%) | 48.5 | 50.5 | 53.9 |
| Married (%) | 76.2 | 78.4 | 78.4 |
| Primary education (%) | 48.0 | 53.4 | 34.3 |
| BMI (kg/m^2^; mean SD) | 32.6 (3.5) | 32.5 (3.4) | 33.5 (3.4) |
| Current smoker (%) | 11.5 | 12.1 | 9.8 |
| Diabetes (%) | 26.6 | 30.4 | 46.1 |
| Adherence to the er-MedDiet (mean, SD) | 8.5 (2.7) | 7.8 (2.5) | 7.4 (2.6) |
| Physical activity (METs/min-week; mean, SD) | 2618 (2332) | 2361 (2037) | 2470 (2133) |
| Total energy intake (kcal/d; mean, SD) | 2406 (615) | 2406 (584) | 2340 (711) |
| ^1^Obtained from Martínez-González et al, *International Journal of Epidemiology*, 2018; except the variable type 2 diabetes that was obtained from Salas-Salvadó et al, *Diabetes Care*, 2018. | | | |

| Supplementary Table 2. Sex differences in baseline cognitive performance. | | | | | | | | |
| --- | --- | --- | --- | --- | --- | --- | --- | --- |
|  | **Men (n=47)** | **Women (n=55)** | **Unadjusted differences** *(Ref= Men)* | | | | **Adjusted* differences** *(Ref= Men)* | |
| **Measure** | **Mean (SD)** | **Mean (SD)** | **Cohen's d** | **β (95%CI)** | **R^2^ (%)** | **P-value** | **β (95%CI)** | **P-value** |
| ***Global cognition composite (Z score)*** | 0.10 (0.50) | -0.11 (0.49) | -0.42 | -0.21 (-0.40, -0.02) | 3.4 | **0.036** | -0.06 (-0.26, 0.13) | 0.526 |
| ***Memory composite (Z score)*** | 0.03 (0.88) | -0.09 (0.63) | -0.15 | -0.12 (-0.41, 0.18) | 0.4 | 0.445 | -0.07 (-0.39, 0.25) | 0.677 |
| RAVTL IR | 7.9 (2.8) | 8.3 (2.4) | 0.17 | 0.4 (-0.6, 1.5) | 0.3 | 0.410 | 0.3 (-0.8, 1.5) | 0.561 |
| RAVTL DR | 7.3 (2.6) | 8.5 (2.2) | 0.49 | 1.2 (0.2, 2.1) | 4.8 | **0.016** | 1.1 (0.0, 2.1) | 0.055 |
| ROCF IR | 19.4 (6.3) | 15.1 (5.0) | -0.76 | -4.3 (-6.6, -2.0) | 11.9 | **<0.001** | -3.0 (-5.4, -0.6) | **0.016** |
| ROCF DR | 19.2 (6.6) | 15.1 (5.2) | -0.70 | -4.1 (-6.5, -1.7) | 10.0 | **<0.001** | -3.2 (-5.6, -0.7) | **0.013** |
| ROCF recognition | 19.3 (3.1) | 18.9 (2.2) | -0.15 | -0.4 (-1.4, 0.6) | 0.5 | 0.463 | -0.5 (-1.7, 0.7) | 0.442 |
| ***Executive functions composite (Z score)*** | 0.12 (0.47) | -0.13 (0.53) | -0.49 | -0.25 (-0.45, -0.05) | 4.8 | **0.015** | -0.03 (-0.23, 0.16) | 0.736 |
| ROCF copy^1^ | 32.1 (3.9) | 31.4 (4.4) | -0.17 | -0.2 (-0.5, 0.2) | 0.3 | 0.406 | 0.1 (-0.3, 0.5) | 0.762 |
| SDMT | 44.5 (13.0) | 36.3 (11.1) | -0.68 | -8.2 (-12.8, -3.5) | 9.7 | **<0.001** | -4.4 (-9.2, 0.5) | 0.083 |
| Stroop interference | -1.5 (8.4) | -1.1 (9.4) | 0.05 | 0.4 (-3.1, 3.9) | 0.9 | 0.814 | 3.0 (-0.9, 7.0) | 0.134 |
| IGT | 14.8 (23.4) | 2.0 (23.0) | -0.55 | -12.8 (-22.0, -3.5) | 6.2 | **0.008** | -9.7 (-20.2, 0.7) | 0.071 |
| CPT commissions^2^ | 26.1 (18.4) | 20.8 (11.4) | -0.35 | -5.3 (-11.2, 0.6) | 2.1 | 0.082 | -6.7 (-13.4, 0.1) | 0.057 |
| CPT omissions^1,2^ | 4.1 (14.4) | 10.4 (25.4) | 0.30 | 0.4 (0.0, 0.7) | 3.5 | **0.034** | 0.1 (-0.3, 0.5) | 0.620 |
| CPT HRT^2^ | 453.5 (71.5) | 480.3 (116.8) | 0.27 | 26.8 (-11.8, 65.3) | 0.9 | 0.177 | 17.0 (-28.6, 62.5) | 0.467 |
| RAVLT= Rey Auditory-Verbal Learning Test; IR=immediate recall; DR=delayed recall; ROCF= Rey-Osterrieth Complex Figure Test; IGT= Iowa Gambling Task; SDMT= Symbol Digit Modalities Test; CPT=Conner’s Continuous Performance Test; HRT= hit reaction time, R^2^=R-squared. ^*^Linear models adjusted by *APOE*-ε4 genotype, age, years of education, diabetes, use of tranquilizers or sedatives, use of lipid-lowering agents, baseline MedDiet adherence and baseline physical activity. ^1^Ordered quantile normalization transformation was applied to estimate sex differences in the ROCF copy and CPT omissions, and to create Z scores of these tests to include them in the executive functioning and global cognition composites. ^2^Higher scores in the CPT indicate lower cognitive performance. Cohen’s d indicates the effect size of sex differences (women vs. men), with cut-offs for interpretation of very small (Cohen's d< 0.2); small (Cohen's d (0.2-0.5)); medium (Cohen's d (0.5-0.8)); large (Cohen's d (0.8-1.2)); and very large (Cohen's d≥ 1.2). A positive value of the Cohen’s d effect size indicates the effect is in favor of women, except for CPT scores.  R^2^ indicates the total explained variance (%) of sex on cognitive performance in crude (unadjusted) linear regression models. A positive value of the estimate (β, 95%CI) of sex differences indicates the effect is in favor of women, except for the CPT scores. Bold values denote statistical significance at the p < 0.05 level | | | | | | | | |

| Supplementary Table 3. Sex differences in cognitive change after 1 and 3 years. | | | | | | | | | | | | |
| --- | --- | --- | --- | --- | --- | --- | --- | --- | --- | --- | --- | --- |
|  |  | **Men (n=47)** | | | **Women (n=55)** | | | **Unadjusted differences*** *(Ref=Men)* | | | **Adjusted differences*** *(Ref=Men)* | |
| **Measure** | **Time** | **Mean change (95%CI)** | **Cohen's d** | **P-value^#^** | **Mean change (95%CI)** | **Cohen's d** | **P-value^#^** | **Cohen's d** | **β (95%CI)** | **P-value** | **β (95%CI)** | **P-value** |
| **Global cognition composite (Z score)** | 1 year | 0.18 (0.10, 0.26) | 0.32 | **<0.001** | 0.11 (0.00, 0.21) | 0.26 | **0.048** | -0.23 | -0.11 (-0.22, 0.00) | 0.059 | -0.11 (-0.24, 0.02) | 0.113 |
|  | 3 years | 0.22 (0.08, 0.36) | 0.27 | **0.011** | 0.18 (0.01, 0.35) | 0.26 | 0.327 | -0.08 | -0.13 (-0.30, 0.04) | 0.147 | -0.23 (-0.44, -0.03) | **0.028** |
| **Memory composite (Z score)** | 1 year | 0.42 (0.24, 0.59) | 0.42 | **<0.001** | 0.25 (0.08, 0.41) | 0.40 | **0.018** | -0.31 | -0.20 (-0.42, 0.01) | 0.067 | -0.17 (-0.44, 0.10) | 0.222 |
|  | 3 years | 0.51 (0.14, 0.88) | 0.40 | 0.067 | 0.19 (-0.05, 0.42) | 0.25 | 0.826 | -0.35 | -0.30 (-0.61, 0.00) | **0.051** | -0.47 (-0.84, -0.10) | **0.017** |
| RAVTL IR | 1 year | 0.4 (-0.4, 1.2) | 0.05 | 0.235 | 0.6 (0.0, 1.3) | 0.21 | 0.230 | 0.09 | 0.5 (-0.4, 1.3) | 0.304 | 0.7 (-0.3, 1.8) | 0.187 |
|  | 3 years | 0.0 (-1.1, 1.2) | 0.10 | 0.805 | -0.4 (-1.4, 0.6) | -0.11 | 0.250 | -0.12 | -0.3 (-1.4, 0.8) | 0.607 | -1.1 (-2.5, 0.3) | 0.139 |
| RAVTL DR | 1 year | 0.7 (0.1, 1.3) | 0.13 | **0.040** | 0.7 (0.0, 1.4) | 0.25 | 0.079 | -0.01 | 0.4 (-0.6, 1.4) | 0.427 | 0.8 (-0.5, 2.0) | 0.230 |
|  | 3 years | 0.5 (-0.8, 1.8) | 0.32 | 0.502 | -0.7 (-1.8, 0.4) | -0.27 | **0.011** | -0.35 | -0.9 (-2.1, 0.3) | 0.151 | -2.0 (-3.4, -0.6) | **0.007** |
| RCFT IR | 1 year | 3.1 (1.8, 4.5) | 0.37 | **<0.001** | 0.6 (-1.1, 2.2) | 0.20 | 0.539 | -0.51 | -3.6 (-5.9, -1.3) | **0.003** | -3.7 (-6.5, -0.8) | **0.013** |
|  | 3 years | 2.5 (0.3, 4.7) | 0.15 | 0.204 | 2.1 (-0.1, 4.3) | 0.30 | 0.425 | -0.06 | -1.1 (-3.4, 1.1) | 0.322 | -3.0 (-5.4, -0.5) | **0.023** |
| RCFT DR | 1 year | 3.0 (1.8, 4.3) | 0.32 | **<0.001** | 1.7 (0.5, 2.8) | 0.37 | **0.012** | -0.35 | -2.0 (-3.8, -0.3) | **0.027** | -2.1 (-4.3, 0.0) | 0.055 |
|  | 3 years | 3.2 (0.9, 5.5) | 0.20 | **0.005** | 3.2 (0.4, 6.1) | 0.41 | 0.134 | 0.00 | -0.8 (-3.2, 1.6) | 0.516 | -3.2 (-5.9, -0.5) | **0.023** |
| RCFT recognition | 1 year | 1.1 (0.2, 2.1) | 0.49 | **0.008** | 0.8 (0.2, 1.5) | 0.28 | **0.022** | -0.12 | -0.9 (-1.7, -0.1) | **0.040** | -0.6 (-1.7, 0.4) | 0.243 |
|  | 3 years | 1.6 (0.3, 2.9) | 0.45 | **0.006** | 1.6 (0.6, 2.7) | 0.56 | **0.002** | 0.01 | -0.1 (-1.2, 0.9) | 0.814 | 0.4 (-0.9, 1.7) | 0.585 |
| **Executive functions composite (Z score)** | 1 year | 0.04 (-0.06, 0.13) | 0.12 | 0.157 | 0.02 (-0.11, 0.15) | 0.12 | 0.618 | -0.05 | -0.07 (-0.20, 0.05) | 0.256 | -0.09 (-0.23, 0.05) | 0.227 |
|  | 3 years | 0.07 (-0.04, 0.19) | 0.07 | 0.194 | 0.19 (0.02, 0.36) | 0.18 | 0.103 | 0.25 | -0.05 (-0.21, 0.12) | 0.600 | -0.09 (-0.29, 0.10) | 0.346 |
| RCFT copy^1^ | 1 year | 1.3 (0.6, 2.0) | 0.34 | 0.966 | 1.1 (0.1, 2.1) | 0.28 | 0.780 | -0.08 | -0.1 (-0.4, 0.3) | 0.734 | 0.0 (-0.4, 0.3) | 0.867 |
|  | 3 years | 0.0 (-1.8, 1.7) | -0.06 | 0.797 | 0.6 (-1.5, 2.7) | -0.05 | 0.524 | 0.10 | 0.0 (-0.5, 0.4) | 0.893 | 0.1 (-0.4, 0.5) | 0.772 |
| SDMT | 1 year | 0.8 (-1.3, 3.0) | 0.14 | 0.549 | 1.8 (0.2, 3.4) | 0.16 | **0.043** | 0.16 | -0.5 (-3.1, 2.2) | 0.722 | -0.5 (-3.5, 2.6) | 0.764 |
|  | 3 years | -3.2 (-7.7, 1.3) | -0.21 | 0.278 | -0.3 (-2.1, 1.6) | -0.04 | 0.937 | 0.31 | 1.0 (-2.8, 4.8) | 0.591 | -1.7 (-5.9, 2.5) | 0.435 |
| Stroop Interference | 1 year | 2.8 (0.2, 5.3) | 0.27 | 0.065 | -0.8 (-3.2, 1.6) | -0.11 | 0.631 | -0.43 | -3.0 (-5.7, -0.3) | **0.032** | -3.2 (-6.7, 0.2) | 0.070 |
|  | 3 years | 1.3 (-1.5, 4.0) | 0.33 | 0.082 | -1.7 (-4.8, 1.3) | -0.18 | 0.218 | -0.34 | -3.6 (-6.5, -0.6) | **0.020** | -3.9 (-7.4, -0.4) | **0.031** |
| IGT | 1 year | 3.0 (-4.9, 11.0) | 0.02 | 0.660 | 6.6 (-4.3, 17.5) | 0.33 | 0.090 | 0.11 | -4.1 (-15.2, 7.0) | 0.468 | 2.7 (-10.3, 15.7) | 0.689 |
|  | 3 years | 4.5 (-8.2, 17.2) | -0.08 | 0.587 | 12.5 (0.5, 24.4) | 0.29 | **0.045** | 0.22 | -3.1 (-14.3, 8.2) | 0.596 | -5.5 (-19.0, 8.0) | 0.431 |
| CPT commissions^2^ | 1 year | 1.0 (-4.9, 6.9) | -0.06 | 0.325 | -1.3 (-5.7, 3.1) | 0.07 | 0.619 | -0.14 | -2.6 (-7.6, 2.3) | 0.302 | -0.2 (-6.4, 6.1) | 0.961 |
|  | 3 years | -5.9 (-11.6, -0.2) | -0.06 | 0.217 | 1.3 (-4.9, 7.6) | 0.19 | 0.436 | 0.40 | 3.9 (-2.7, 10.5) | 0.255 | -1.8 (-9.9, 6.3) | 0.667 |
| CPT omissions^1,2^ | 1 year | -3.4 (-8.6, 1.9) | -0.30 | 0.732 | -5.7 (-12.4, 0.9) | -0.45 | 0.308 | -0.12 | 0.2 (-0.1, 0.5) | 0.238 | 0.2 (-0.2, 0.6) | 0.237 |
|  | 3 years | -2.0 (-5.4, 1.4) | -0.27 | 0.967 | -5.5 (-12.5, 1.6) | -0.36 | 0.519 | -0.21 | -0.2 (-0.6, 0.2) | 0.365 | -0.3 (-0.8, 0.1) | 0.159 |
| CPT HRT^2^ | 1 year | -1.1 (-18.5, 16.4) | 0.06 | 0.981 | 23.4 (-39.4, 86.3) | -0.06 | 0.693 | 0.15 | 15.5 (-17.1, 48.2) | 0.353 | 8.9 (-32.0, 49.9) | 0.671 |
|  | 3 years | -22.6 (-57.7, 12.5) | -0.39 | **0.032** | -55.7 (-103.6, -7.8) | -0.56 | **0.008** | -0.26 | -23.5 (-68.5, 21.6) | 0.311 | -9.3 (-65.7, 47.0) | 0.747 |
| The rates of missing values in cognitive outcomes were 19.1% (n=9) in men and 16.4% (n=9) in women after 1 year; and 29.8% (n=14) in men and 30.9% (n=17) in women after 3 years. Inverse probability weights were applied to compute 1 year and 3 years mean change and P-values. RAVLT= Rey Auditory-Verbal Learning Test; IR=immediate recall; DR=delayed recall; ROCF= Rey-Osterrieth Complex Figure Test; IGT= Iowa Gambling Task; SDMT= Symbol Digit Modalities Test; CPT=Conner’s Continuous Performance Test; HRT= hit reaction time. ^1^Ordered quantile normalization transformation was applied to estimate sex differences in the ROCF copy and CPT omissions, and to create Z scores of these tests to include them in the executive functioning and global cognition composites. ^2^Higher scores in the CPT indicate lower cognitive performance. The effect size of cognitive changes in men and women, and of differences in cognitive change between men and women, was estimated with the Cohen’s d, with cut-offs for interpretation of very small (Cohen's d< 0.2); small (Cohen's d (0.2-0.5)); medium (Cohen's d (0.5-0.8)); large (Cohen's d (0.8-1.2)); and very large (Cohen's d≥ 1.2). A positive value of the Cohen’s d effect size of sex differences indicates the effect is in favor of women, except for CPT scores. ^#^Linear mixed effects models were used to analyze cognitive changes by sex, and were adjusted by *APOE*-ε4 genotype, age, years of education, diabetes, use of tranquilizers or sedatives, use of lipid-lowering agents, baseline MedDiet adherence and baseline physical activity. ^*^Analysis of covariance (ANCOVA) models were used to analyze sex differences in cognitive change with respect to baseline. These models used as outcome variables the changes from baseline to 1 year or 3 years, and included sex as an independent variable adjusting for the baseline score (unadjusted or crude models). Adjusted models additionally included *APOE*-ε4 genotype, age, years of education, diabetes, use of tranquilizers or sedatives, use of lipid-lowering agents, baseline MedDiet adherence, and baseline physical activity as covariates. A positive value of the estimate (β, 95%CI) of sex differences indicates the effect is in favor of women, except for the CPT scores. Bold values denote statistical significance at the p < 0.05 level | | | | | | | | | | | | |

| Supplementary Table 4. Differences in baseline cognitive performance by *APOE*-ɛ4 genotype | | | | | |
| --- | --- | --- | --- | --- | --- |
|  | ***APOE*-ɛ4 non-carriers (n=83)** | ***APOE*-ɛ4 carriers (n=19)** | **Differences** *(Ref = APOE-ɛ4 non-carriers)* | | |
| **Measure** | **Mean (SD)** | **Mean (SD)** | **Cohen's d** | **β (95%CI)^*^** | **P-value^*^** |
| ***Global cognition composite (Z score)*** | -0.03 (0.49) | 0.09 (0.55) | 0.25 | 0.06 (-0.19, 0.30) | 0.648 |
| ***Memory composite (Z score)*** | -0.07 (0.74) | 0.09 (0.85) | 0.21 | 0.16 (-0.23, 0.55) | 0.422 |
| RAVTL IR | 8.2 (2.5) | 7.9 (3.0) | -0.12 | 0.0 (-1.4, 1.3) | 0.945 |
| RAVTL DR | 8.1 (2.4) | 7.6 (2.9) | -0.18 | -0.3 (-1.5, 1.0) | 0.691 |
| ROCF IR | 16.7 (5.9) | 18.3 (6.4) | 0.27 | 1.2 (-1.8, 4.2) | 0.430 |
| ROCF DR | 16.6 (6.2) | 18.7 (6.2) | 0.34 | 1.6 (-1.5, 4.7) | 0.319 |
| ROCF recognition | 19.0 (2.8) | 19.6 (2.0) | 0.25 | 0.5 (-0.9, 1.9) | 0.489 |
| ***Executive functions composite (Z score)*** | -0.03 (0.53) | 0.09 (0.47) | 0.24 | 0.02 (-0.23, 0.26) | 0.895 |
| ROCF copy^1^ | 31.6 (4.4) | 32.3 (3.1) | 0.18 | 0.0 (-0.5, 0.5) | 0.991 |
| SDMT | 39.7 (12.9) | 41.6 (11.3) | 0.15 | -0.9 (-6.8, 4.9) | 0.759 |
| Stroop Interference | -1.0 (9.1) | -2.2 (8.3) | -0.13 | -1.5 (-6.1, 3.1) | 0.519 |
| IGT | 7.4 (22.6) | 10.0 (29.3) | 0.11 | -4.5 (-16.3, 7.3) | 0.457 |
| CPT commissions^2^ | 24.2 (15.1) | 19.5 (15.5) | -0.31 | -7.5 (-15.2, 0.1) | 0.057 |
| CPT omissions^1,2^ | 7.6 (21.1) | 6.6 (21.9) | -0.05 | -0.2 (-0.7, 0.2) | 0.357 |
| CPT HRT^2^ | 465.4 (106.5) | 477.7 (54.6) | 0.12 | 23.3 (-28.6, 75.1) | 0.381 |
| RAVLT= Rey Auditory-Verbal Learning Test; IR=immediate recall; DR=delayed recall; ROCF= Rey-Osterrieth Complex Figure Test; IGT= Iowa Gambling Task; SDMT= Symbol Digit Modalities Test; CPT=Conner’s Continuous Performance Test; HRT= hit reaction time. ^*^Linear models adjusted by gender, age, smoking status and use of lipid-lowering agents. ^1^Ordered quantile normalization transformation was applied to estimate sex differences in the ROCF copy and CPT omissions, and to create Z scores of these tests to include them in the executive functioning and global cognition composites. ^2^Higher scores in the CPT indicate lower cognitive performance. Cohen’s d indicates the effect size of APOE differences (ɛ4 carriers vs. non-carriers), with cut-offs for interpretation of very small (Cohen's d< 0.2); small (Cohen's d (0.2-0.5)); medium (Cohen's d (0.5-0.8)); large (Cohen's d (0.8-1.2)); and very large (Cohen's d≥ 1.2). A positive value of the Cohen’s d effect size indicates the effect is in favor of *APOE*-ɛ4 carriers, except for CPT scores. A positive value of the estimate (β, 95%CI) of cognitive differences by *APOE* genotype indicates the effect is in favor of *APOE*-ɛ4 carriers, except for the CPT scores. | | | | | |

| Supplementary Table 5. *APOE*-ɛ4 differences in cognitive change after 1 and 3 years | | | | | | | | | | |
| --- | --- | --- | --- | --- | --- | --- | --- | --- | --- | --- |
|  |  | ***APOE*-ɛ4 non-carriers (n=83)** | | | ***APOE*-ɛ4 carriers (n=19)** | | | **Differences by *APOE* genotype** *(Ref = APOE-ɛ4 non-carriers)* | | |
| **Measure** | **Time** | **Mean change (95%CI)** | **Cohen's d** | **P-value^#^** | **Mean change (95%CI)** | **Cohen's d** | **P-value^#^** | **Cohen's d** | **β (95%CI)^*^** | **P-value^*^** |
| ***Global cognition composite***  ***(Z score)*** | 1 year | 0.12 (0.04, 0.20) | 0.28 | **0.003** | 0.22 (0.10, 0.34) | 0.26 | **<0.001** | 0.33 | 0.13 (-0.02, 0.27) | 0.102 |
|  | 3 years | 0.23 (0.11, 0.35) | 0.27 | **0.019** | 0.00 (-0.18, 0.17) | 0.33 | 0.849 | -0.52 | -0.17 (-0.44, 0.10) | 0.213 |
| ***Memory composite***  ***(Z score)*** | 1 year | 0.29 (0.15, 0.43) | 0.40 | **<0.001** | 0.48 (0.31, 0.65) | 0.39 | **<0.001** | 0.35 | 0.14 (-0.15, 0.44) | 0.351 |
|  | 3 years | 0.40 (0.16, 0.63) | 0.36 | 0.055 | -0.03 (-0.50, 0.45) | 0.24 | 0.671 | -0.46 | -0.33 (-0.81, 0.15) | 0.184 |
| RAVTL IR | 1 year | 0.5 (-0.1, 1.1) | 0.12 | 0.207 | 0.9 (0.0, 1.7) | 0.14 | 0.086 | 0.18 | 0.2 (-1.0, 1.4) | 0.727 |
|  | 3 years | -0.1 (-0.9, 0.8) | 0.00 | 0.454 | -1.0 (-2.5, 0.5) | -0.05 | 0.447 | -0.28 | -0.9 (-2.7, 0.9) | 0.321 |
| RAVTL DR | 1 year | 0.6 (0.0, 1.1) | 0.18 | 0.060 | 1.2 (0.4, 2.0) | 0.23 | **0.015** | 0.30 | 0.6 (-0.7, 1.9) | 0.375 |
|  | 3 years | 0.0 (-1.0, 0.9) | 0.01 | 0.296 | -1.0 (-2.6, 0.5) | 0.06 | 0.448 | -0.29 | -1.0 (-2.9, 0.9) | 0.327 |
| ROCF IR | 1 year | 1.2 (-0.1, 2.4) | 0.21 | 0.120 | 4.0 (2.0, 6.0) | 0.44 | **0.002** | 0.57 | 1.5 (-1.5, 4.4) | 0.328 |
|  | 3 years | 2.6 (1.0, 4.3) | 0.26 | 0.094 | 0.4 (-2.9, 3.8) | 0.21 | 0.956 | -0.34 | -2.4 (-5.8, 1.0) | 0.169 |
| ROCF DR | 1 year | 2.0 (1.0, 2.9) | 0.33 | **<0.001** | 3.7 (1.6, 5.7) | 0.36 | **0.007** | 0.43 | 1.0 (-1.3, 3.2) | 0.400 |
|  | 3 years | 3.8 (1.8, 5.7) | 0.36 | **<0.001** | 0.1 (-3.1, 3.3) | 0.22 | 0.798 | -0.49 | -2.7 (-6.4, 1.0) | 0.158 |
| ROCF recognition | 1 year | 1.0 (0.4, 1.6) | 0.35 | **0.001** | 0.9 (-0.3, 2.1) | 0.54 | **0.013** | -0.03 | 0.4 (-0.8, 1.5) | 0.519 |
|  | 3 years | 1.6 (0.7, 2.6) | 0.50 | **0.002** | 1.5 (0.3, 2.7) | 0.73 | **0.005** | -0.04 | -0.3 (-1.9, 1.4) | 0.750 |
| ***Executive functions composite (Z score)*** | 1 year | 0.03 (-0.07, 0.12) | 0.12 | 0.435 | 0.04 (-0.14, 0.21) | 0.04 | 0.186 | 0.03 | 0.09 (-0.07, 0.26) | 0.259 |
|  | 3 years | 0.15 (0.04, 0.27) | 0.13 | **0.048** | 0.01 (-0.17, 0.19) | 0.29 | 0.749 | -0.33 | -0.02 (-0.28, 0.23) | 0.850 |
| ROCF copy^1^ | 1 year | 1.1 (0.4, 1.9) | 0.31 | 0.552 | 1.4 (0.3, 2.5) | 0.22 | 0.426 | 0.10 | 0.3 (-0.2, 0.7) | 0.240 |
|  | 3 years | 0.9 (-0.5, 2.3) | 0.04 | 0.380 | -3.1 (-5.9, -0.4) | -0.74 | 0.119 | -0.74 | -0.8 (-1.5, -0.2) | **0.010** |
| SDMT | 1 year | 1.4 (-0.1, 2.9) | 0.15 | 0.078 | 1.3 (-0.8, 3.4) | 0.02 | 0.411 | -0.02 | 0.7 (-2.7, 4.1) | 0.682 |
|  | 3 years | -1.7 (-4.4, 1.0) | -0.15 | 0.341 | -1.2 (-4.5, 2.1) | 0.17 | 0.351 | 0.05 | 1.1 (-4.2, 6.5) | 0.680 |
| Stroop Interference | 1 year | 0.0 (-1.9, 1.9) | -0.02 | 0.923 | 4.1 (-0.5, 8.6) | 0.33 | 0.209 | 0.49 | 1.9 (-1.9, 5.6) | 0.331 |
|  | 3 years | -0.9 (-3.2, 1.4) | -0.05 | 0.611 | 3.2 (0.3, 6.1) | 0.73 | **0.036** | 0.49 | 3.6 (-1.1, 8.2) | 0.138 |
| IGT | 1 year | 4.5 (-3.5, 12.6) | 0.19 | 0.207 | 7.4 (-6.6, 21.4) | 0.13 | 0.379 | 0.09 | 0.9 (-13.9, 15.7) | 0.908 |
|  | 3 years | 11.2 (1.9, 20.5) | 0.14 | 0.286 | -6.7 (-20.0, 6.5) | 0.00 | 0.517 | -0.53 | -6.2 (-23.4, 11.0) | 0.484 |
| CPT commissions^2^ | 1 year | -1.9 (-5.2, 1.4) | -0.10 | 0.566 | 6.9 (-4.5, 18.4) | 0.30 | 0.491 | 0.55 | 4.4 (-1.9, 10.7) | 0.174 |
|  | 3 years | -3.5 (-8.4, 1.3) | 0.02 | 0.318 | 5.6 (-1.6, 12.9) | 0.04 | 0.068 | 0.52 | 3.9 (-6.6, 14.3) | 0.471 |
| CPT omissions^1,2^ | 1 year | -5.7 (-11.0, -0.4) | -0.40 | 0.810 | -0.1 (-0.6, 0.3) | -0.32 | **0.043** | 0.28 | 0.1 (-0.3, 0.6) | 0.518 |
|  | 3 years | -2.8 (-6.3, 0.7) | -0.32 | 0.969 | -9.8 (-28.8, 9.2) | -0.35 | 0.653 | -0.40 | -0.4 (-1.0, 0.1) | 0.145 |
| CPT HRT^2^ | 1 year | 19.8 (-23.6, 63.2) | 0.05 | 0.510 | -19.9 (-45.2, 5.4) | -0.42 | 0.171 | -0.24 | -23.6 (-68.9, 21.6) | 0.310 |
|  | 3 years | -32.4 (-66.2, 1.3) | -0.45 | **0.003** | -82.3 (-131.0, -33.5) | -0.59 | **0.005** | -0.41 | -6.5 (-76.9, 64.0) | 0.858 |
| The rates of missing values in cognitive outcomes were 18.1% (n=15) in *APOE*-ɛ4 non-carriers and 15.8% (n=3) in *APOE*-ɛ4 carriers after 1 year; and 28.9% (n=24) in *APOE*-ɛ4 non-carriers and 36.8% (n=7) in *APOE*-ɛ4 carriers after 3 years. RAVLT= Rey Auditory-Verbal Learning Test; IR=immediate recall; DR=delayed recall; ROCF= Rey-Osterrieth Complex Figure Test; IGT= Iowa Gambling Task; SDMT= Symbol Digit Modalities Test; CPT=Conner’s Continuous Performance Test; HRT= hit reaction time. ^1^Ordered quantile normalization transformation was applied to estimate sex differences in the ROCF copy and CPT omissions, and to create Z scores of these tests to include them in the executive functioning and global cognition composites. ^2^Higher scores in the CPT indicate lower cognitive performance. The effect size of cognitive changes in *APOE*-ɛ4 carriers and non-carriers, and of differences in cognitive change between *APOE* genotypes, was estimated with the Cohen’s d, with cut-offs for interpretation of very small (Cohen's d< 0.2); small (Cohen's d (0.2-0.5)); medium (Cohen's d (0.5-0.8)); large (Cohen's d (0.8-1.2)); and very large (Cohen's d≥ 1.2). A positive value of the Cohen’s d effect size of *APOE* differences indicates the effect is in favor of *APOE*- ɛ4 carriers, except for CPT scores. ^#^Linear mixed effects models were used to analyze cognitive changes by *APOE* genotype, and were adjusted by gender, age, smoking status and use of lipid-lowering agents. ^*^ANCOVA models were used to analyze *APOE* differences in cognitive change with respect to baseline. These models used as outcome variables the changes from baseline to 1 year or 3 years, and included *APOE* genotype as an independent variable adjusting for the baseline score, gender, age, smoking status and use of lipid-lowering agents. A positive value of modeled differences (β, 95%CI) indicates the effect is in favor of *APOE*-ɛ4 carriers, except for the CPT scores. Bold values denote statistical significance at the p < 0.05 level. | | | | | | | | | | |

| Supplementary Table 6. Differences in baseline concentrations of eCBs and NAEs by *APOE*-ɛ4 genotype | | | | | | |
| --- | --- | --- | --- | --- | --- | --- |
|  | **APOE-ɛ4 non-carriers (n=83)** | | **APOE-ɛ4 carriers (n=19)** | | **Differences by *APOE* genotype** *(Ref = APOE-ɛ4 non-carriers)* | |
| **Compound** | **Mean (SD)** | **Median (Q1, Q3)** | **Mean (SD)** | **Median (Q1, Q3)** | **Cohen's d** | **P-value*** |
| 2-AG (nM) | 4.70 (2.97) | 3.83 (3.13, 5.46) | 5.12 (4.37) | 3.63 (3.03, 5.44) | 0.13 | 0.990 |
| AEA (nM) | 0.78 (0.25) | 0.76 (0.59, 0.92) | 0.74 (0.18) | 0.70 (0.64, 0.84) | -0.16 | 0.633 |
| OEA (nM) | 10.43 (2.55) | 10.14 (8.84, 12.46) | 9.74 (2.31) | 9.69 (7.91, 11.57) | -0.28 | 0.552 |
| PEA (nM) | 18.45 (3.95) | 18.74 (15.61, 21.49) | 18.17 (3.82) | 17.03 (15.89, 19.13) | -0.07 | 0.819 |
| DHEA (nM) | 1.22 (0.42) | 1.24 (0.91, 1.44) | 1.26 (0.52) | 1.13 (0.91, 1.51) | 0.09 | 0.365 |
| DEA (nM) | 0.18 (0.05) | 0.18 (0.15, 0.21) | 0.18 (0.04) | 0.18 (0.15, 0.20) | -0.07 | 0.516 |
| DGLEA (nM) | 0.16 (0.06) | 0.16 (0.13, 0.19) | 0.15 (0.05) | 0.14 (0.13, 0.17) | -0.23 | 0.851 |
| LEA (nM) | 2.18 (0.67) | 2.11 (1.70, 2.55) | 2.23 (0.72) | 2.14 (1.71, 2.75) | 0.08 | 0.509 |
| POEA (nM) | 1.91 (0.88) | 1.80 (1.17, 2.56) | 1.55 (0.63) | 1.27 (1.08, 2.06) | -0.43 | 0.299 |
| SEA (nM) | 4.78 (1.10) | 4.63 (3.99, 5.43) | 4.58 (0.83) | 4.35 (3.96, 5.06) | -0.19 | 0.955 |
| OEA/AEA | 13.84 (2.34) | 13.37 (12.24, 15.51) | 13.39 (2.16) | 13.60 (12.40, 14.79) | -0.20 | 0.830 |
| OEA/PEA | 0.56 (0.07) | 0.56 (0.52, 0.61) | 0.54 (0.09) | 0.54 (0.48, 0.60) | -0.38 | 0.199 |
| PEA/AEA | 24.71 (4.41) | 24.21 (22.18, 27.06) | 25.22 (4.19) | 24.81 (22.92, 26.79) | 0.12 | 0.349 |
| DHEA/AEA | 1.65 (0.56) | 1.63 (1.25, 1.93) | 1.72 (0.54) | 1.57 (1.37, 2.06) | 0.12 | 0.379 |
| ^*^Linear models adjusted by gender, age, smoking status and use of lipid-lowering agents. Cohen’s d indicates the effect size of APOE differences (ɛ4 carriers vs. non-carriers) in baseline concentrations of eCBs and NAEs, with cut-offs for interpretation of very small (Cohen's d< 0.2); small (Cohen's d (0.2-0.5)); medium (Cohen's d (0.5-0.8)); large (Cohen's d (0.8-1.2)); and very large (Cohen's d≥ 1.2). A positive value of the Cohen’s d effect size or the estimate (β, 95%CI) indicates higher values in *APOE*-ɛ4 carriers. | | | | | | |

| Supplementary Table 7. Changes in eCBs and NAEs concentrations by APOE-ɛ4 genotype | | | | | | | | | | |
| --- | --- | --- | --- | --- | --- | --- | --- | --- | --- | --- |
|  |  | **APOE-ɛ4 non-carriers (n=83)** | | | **APOE-ɛ4 carriers (n=19)** | | | **Differences (Ref = APOE-ɛ4 non-carriers)** | | |
| **Compound** | **Time** | **Mean change (95% CI)** | **Cohen's d** | **P-value^#^** | **Mean change (95% CI)** | **Cohen's d** | **P-value^#^** | **Cohen's d** | **β (95% CI)^*^** | **P-value^*^** |
| 2-AG (nM) | 6 months | -0.81 (-1.31, -0.32) | -0.30 | **0.002** | -0.90 (-2.56, 0.77) | -0.24 | 0.272 | -0.06 | 0.03 (-0.97, 1.02) | 0.961 |
|  | 1 year | -0.71 (-1.38, -0.04) | -0.31 | **0.025** | -0.02 (-2.37, 2.34) | -0.16 | 0.656 | 0.39 | 0.44 (-1.24, 2.12) | 0.607 |
|  | 3 years | -0.76 (-1.42, -0.10) | -0.30 | **0.033** | -0.16 (-1.12, 0.81) | -0.33 | 0.658 | 0.38 | 0.20 (-0.76, 1.16) | 0.685 |
| AEA (nM) | 6 months | -0.09 (-0.13, -0.04) | -0.34 | **<0.001** | 0.07 (-0.07, 0.20) | 0.23 | 0.317 | 0.34 | 0.15 (0.05, 0.25) | **0.003** |
|  | 1 year | -0.03 (-0.09, 0.02) | -0.09 | 0.315 | 0.08 (-0.03, 0.18) | 0.27 | 0.266 | 0.23 | 0.08 (-0.04, 0.20) | 0.178 |
|  | 3 years | 0.05 (-0.02, 0.12) | 0.20 | 0.136 | 0.12 (-0.01, 0.25) | 0.53 | 0.080 | 0.13 | 0.09 (-0.05, 0.23) | 0.234 |
| OEA (nM) | 6 months | -0.69 (-1.13, -0.25) | -0.27 | **0.003** | 0.86 (-0.38, 2.11) | 0.30 | 0.162 | 1.08 | 1.37 (0.36, 2.38) | **0.009** |
|  | 1 year | 0.14 (-0.42, 0.71) | 0.11 | 0.439 | 1.28 (0.18, 2.38) | 0.42 | 0.041 | 0.74 | 0.53 (-0.70, 1.77) | 0.399 |
|  | 3 years | 0.81 (0.11, 1.50) | 0.32 | **0.016** | 1.03 (0.00, 2.07) | 0.38 | 0.066 | 0.14 | 0.21 (-1.27, 1.68) | 0.786 |
| PEA (nM) | 6 months | -1.71 (-2.39, -1.04) | -0.44 | **<0.001** | 0.25 (-1.74, 2.24) | 0.06 | 0.795 | 1.09 | 2.04 (0.55, 3.53) | **0.009** |
|  | 1 year | -0.19 (-1.02, 0.63) | -0.03 | 0.711 | 1.12 (-0.31, 2.54) | 0.00 | 0.187 | 0.71 | 0.54 (-1.32, 2.40) | 0.570 |
|  | 3 years | 0.05 (-0.91, 1.01) | 0.01 | 0.926 | 0.49 (-0.84, 1.83) | -0.03 | 0.548 | 0.23 | 0.56 (-1.37, 2.49) | 0.570 |
| DHEA (nM) | 6 months | -0.08 (-0.14, -0.01) | -0.19 | **0.034** | 0.00 (-0.20, 0.20) | 0.01 | 0.977 | 0.14 | 0.11 (-0.04, 0.25) | 0.148 |
|  | 1 year | 0.03 (-0.05, 0.12) | 0.09 | 0.425 | 0.02 (-0.16, 0.19) | -0.15 | 0.951 | -0.03 | -0.03 (-0.23, 0.17) | 0.790 |
|  | 3 years | 0.04 (-0.04, 0.11) | 0.13 | 0.312 | -0.04 (-0.22, 0.14) | -0.34 | 0.453 | -0.14 | -0.09 (-0.26, 0.07) | 0.266 |
| DEA (nM) | 6 months | -0.02 (-0.03, -0.02) | -0.47 | **<0.001** | 0.01 (-0.02, 0.03) | 0.09 | 0.690 | 0.14 | 0.03 (0.01, 0.05) | **0.006** |
|  | 1 year | -0.01 (-0.02, 0.00) | -0.16 | 0.075 | 0.01 (-0.01, 0.03) | 0.12 | 0.248 | 0.11 | 0.01 (-0.01, 0.04) | 0.196 |
|  | 3 years | 0.00 (-0.01, 0.01) | 0.05 | 0.774 | 0.02 (-0.01, 0.04) | 0.28 | 0.226 | 0.07 | 0.02 (-0.01, 0.04) | 0.135 |
| DGLEA (nM) | 6 months | -0.01 (-0.02, -0.01) | -0.25 | **0.002** | 0.00 (-0.01, 0.02) | 0.10 | 0.613 | 0.10 | 0.02 (0.00, 0.04) | **0.048** |
|  | 1 year | 0.00 (-0.01, 0.01) | -0.06 | 0.483 | 0.01 (0.00, 0.03) | 0.22 | 0.088 | 0.08 | 0.01 (-0.01, 0.03) | 0.238 |
|  | 3 years | 0.00 (-0.01, 0.01) | 0.05 | 0.664 | 0.01 (-0.01, 0.04) | 0.27 | 0.329 | 0.04 | 0.01 (-0.01, 0.03) | 0.332 |
| LEA (nM) | 6 months | -0.21 (-0.33, -0.09) | -0.34 | **<0.001** | 0.07 (-0.28, 0.43) | 0.10 | 0.668 | 0.38 | 0.36 (0.11, 0.61) | **0.007** |
|  | 1 year | -0.05 (-0.18, 0.09) | -0.06 | 0.530 | 0.23 (-0.04, 0.51) | 0.09 | 0.182 | 0.37 | 0.21 (-0.07, 0.48) | 0.144 |
|  | 3 years | 0.07 (-0.10, 0.24) | 0.11 | 0.430 | 0.24 (0.00, 0.48) | 0.22 | 0.061 | 0.21 | 0.23 (-0.12, 0.57) | 0.210 |
| POEA (nM) | 6 months | -0.23 (-0.38, -0.09) | -0.26 | **0.003** | -0.02 (-0.24, 0.20) | -0.03 | 0.850 | 0.27 | 0.13 (-0.15, 0.41) | 0.357 |
|  | 1 year | -0.07 (-0.25, 0.10) | -0.06 | 0.470 | 0.13 (-0.07, 0.32) | 0.04 | 0.258 | 0.24 | -0.05 (-0.40, 0.29) | 0.767 |
|  | 3 years | -0.01 (-0.22, 0.20) | -0.03 | 0.823 | 0.00 (-0.22, 0.22) | 0.00 | 0.909 | 0.01 | 0.03 (-0.42, 0.47) | 0.913 |
| SEA (nM) | 6 months | -0.47 (-0.66, -0.28) | -0.44 | **<0.001** | -0.16 (-0.43, 0.12) | -0.19 | 0.252 | 0.35 | 0.30 (-0.06, 0.66) | 0.101 |
|  | 1 year | -0.01 (-0.23, 0.21) | 0.00 | 0.941 | 0.18 (-0.16, 0.52) | -0.06 | 0.401 | 0.20 | 0.06 (-0.45, 0.57) | 0.819 |
|  | 3 years | -0.03 (-0.27, 0.21) | 0.02 | 0.922 | 0.22 (-0.13, 0.56) | 0.11 | 0.263 | 0.25 | 0.18 (-0.33, 0.69) | 0.493 |
| OEA/AEA | 6 months | 0.80 (0.40, 1.21) | 0.31 | **<0.001** | 0.32 (-0.38, 1.02) | 0.15 | 0.351 | -0.37 | -0.84 (-1.70, 0.01) | 0.056 |
|  | 1 year | 0.96 (0.45, 1.46) | 0.37 | **<0.001** | 0.35 (-0.51, 1.21) | 0.17 | 0.365 | -0.42 | -1.04 (-2.18, 0.11) | 0.079 |
|  | 3 years | 0.25 (-0.26, 0.77) | 0.11 | 0.363 | -0.18 (-1.15, 0.78) | -0.18 | 0.640 | -0.30 | -0.78 (-1.95, 0.40) | 0.198 |
| OEA/PEA | 6 months | 0.02 (0.01, 0.03) | 0.30 | **0.001** | 0.03 (0.01, 0.05) | 0.40 | **0.002** | 0.06 | 0.01 (-0.02, 0.03) | 0.615 |
|  | 1 year | 0.02 (0.00, 0.03) | 0.32 | **0.007** | 0.04 (0.01, 0.08) | 0.57 | **0.022** | 0.09 | 0.02 (-0.02, 0.05) | 0.294 |
|  | 3 years | 0.04 (0.02, 0.06) | 0.59 | **<0.001** | 0.05 (0.01, 0.08) | 0.63 | **0.015** | 0.02 | 0.00 (-0.04, 0.04) | 0.971 |
| PEA/AEA | 6 months | 0.52 (-0.33, 1.38) | 0.10 | 0.245 | -0.98 (-2.20, 0.24) | -0.24 | 0.107 | -0.79 | -1.80 (-3.54, -0.07) | **0.044** |
|  | 1 year | 0.87 (-0.09, 1.84) | 0.15 | 0.097 | -1.22 (-3.66, 1.22) | -0.45 | 0.287 | -1.02 | -2.39 (-4.53, -0.26) | **0.031** |
|  | 3 years | -1.32 (-2.44, -0.21) | -0.33 | **0.010** | -2.63 (-4.85, -0.41) | -0.81 | 0.017 | -0.62 | -1.56 (-3.76, 0.64) | 0.169 |
| DHEA/AEA | 6 months | 0.09 (0.00, 0.19) | 0.15 | 0.054 | -0.09 (-0.26, 0.08) | -0.19 | 0.270 | -0.29 | -0.21 (-0.42, 0.00) | **0.050** |
|  | 1 year | 0.13 (0.00, 0.25) | 0.19 | 0.060 | -0.13 (-0.36, 0.09) | -0.30 | 0.185 | -0.36 | -0.25 (-0.53, 0.03) | 0.083 |
|  | 3 years | -0.05 (-0.15, 0.06) | -0.05 | 0.359 | -0.24 (-0.44, -0.04) | -0.77 | **0.012** | -0.30 | -0.27 (-0.49, -0.06) | **0.015** |
| The rates of missing values in eCBs were 9.6% (n=8) in APOE-e4 non-carriers and 21.1% (n=4) in APOE4 carriers after 1 year; and 18.1% (n=15) in APOE4 non-carriers and 21.1% (n=4) in APOE4 carriers after 3 years. The effect size of changes within group (*APOE*-ɛ4 carriers and non-carriers), and of differences in the rate of change between groups (*APOE* genotypes), was estimated with the Cohen’s d, with cut-offs for interpretation of very small (Cohen's d< 0.2); small (Cohen's d (0.2-0.5)); medium (Cohen's d (0.5-0.8)); large (Cohen's d (0.8-1.2)); and very large (Cohen's d≥ 1.2). A positive value of the Cohen’s d effect size of *APOE* differences indicates the effect is in favor of *APOE*- ɛ4 carriers. ^#^Linear mixed effects models were used to analyze within-group changes, and were adjusted by gender, age, smoking status and use of lipid-lowering agents. ^*^ANCOVA models were used to analyze between group differences in the rate of change with respect to baseline. These models used as outcome variables the changes from baseline to 6 months, 1 year or 3 years, and included *APOE* genotype as an independent variable adjusting for the baseline score, gender, age, smoking status and use of lipid-lowering agents. A positive value of modeled differences (β, 95%CI) indicates the effect is in favor of *APOE*-ɛ4 carriers. Bold values denote statistical significance at the p < 0.05 level. | | | | | | | | | | |

| Supplementary Table 8. Differences in baseline cardiovascular and lifestyle risk factors by *APOE*-ɛ4 genotype | | | | | | |
| --- | --- | --- | --- | --- | --- | --- |
|  | **APOE-ɛ4 non-carriers (n=83)** | | **APOE-ɛ4 carriers (n=19)** | | **Differences** | |
| **Compound** | **Mean (SD)** | **Median (Q1, Q3)** | **Mean (SD)** | **Median (Q1, Q3)** | **Cohen's d** | **P-value*** |
| MedDiet adherence | 7.4 (2.6) | 7.0 (5.5, 9.0) | 7.1 (2.8) | 7.0 (5.0, 9.0) | -0.12 | 0.477 |
| Physical activity (MET x min/week) | 2489.5 (2204.7) | 1678.3 (909.1, 3356.6) | 2383.9 (1837.0) | 2377.6 (958.0, 3078.3) | -0.05 | 0.764 |
| Weight (kg) | 88.9 (14.9) | 87.6 (76.9, 98.5) | 91.8 (13.2) | 92.5 (83.6, 102.9) | 0.20 | 0.822 |
| BMI (kg/m^2^) | 33.7 (3.5) | 33.5 (30.7, 36.3) | 33.6 (2.9) | 33.7 (31.0, 36.1) | -0.03 | 0.819 |
| Hip (cm) | 114.4 (9.3) | 114.0 (106.2, 121.5) | 113.2 (7.3) | 113.0 (108.0, 118.5) | -0.14 | 0.941 |
| Waist (cm) | 111.1 (10.2) | 110.0 (104.0, 115.5) | 113.6 (9.0) | 117.0 (107.5, 120.8) | 0.24 | 0.819 |
| Systolic BP (mmHg) | 146.0 (14.0) | 145.0 (137.0, 153.5) | 144.6 (13.2) | 144.0 (134.5, 152.5) | -0.10 | 0.748 |
| Diastolic BP (mmHg) | 77.3 (10.6) | 79.0 (71.0, 85.0) | 79.8 (10.2) | 80.0 (77.0, 86.0) | 0.24 | 0.243 |
| HDL-c (mg/dL) | 53.0 (9.8) | 52.0 (47.0, 57.5) | 51.3 (11.9) | 50.0 (42.5, 60.0) | -0.17 | 0.578 |
| LDL-c (mg/dL) | 145.8 (37.1) | 147.0 (121.0, 167.8) | 144.4 (28.7) | 140.5 (128.5, 165.0) | -0.04 | 0.407 |
| Total cholesterol (mg/dL) | 228.6 (43.3) | 228.0 (193.5, 253.5) | 223.6 (30.2) | 217.0 (201.5, 242.0) | -0.12 | 0.542 |
| Triglycerides (mg/dL) | 158.5 (79.1) | 139.0 (106.0, 199.5) | 172.6 (131.1) | 135.0 (87.5, 213.0) | 0.16 | 0.915 |
| HbA1c (%) | 6.3 (1.0) | 6.0 (5.7, 6.6) | 6.5 (0.8) | 6.6 (5.9, 6.9) | 0.16 | 0.849 |
| Glucose (mg/dL) | 118.9 (30.8) | 112.0 (100.0, 133.0) | 132.8 (29.8) | 130.0 (107.0, 152.5) | 0.45 | 0.168 |
| Insulin (µUI/mL) | 9.1 (5.1) | 7.8 (5.9, 11.4) | 8.6 (5.0) | 6.6 (4.6, 11.6) | -0.09 | 0.516 |
| HOMA-IR | 2.7 (1.8) | 2.3 (1.4, 3.4) | 3.0 (2.2) | 2.2 (1.5, 3.4) | 0.14 | 0.939 |
| ^*^Linear models adjusted by gender, age, smoking status and use of lipid-lowering agents. Cohen’s d indicates the effect size of APOE differences (ɛ4 carriers vs. non-carriers) in baseline concentrations of eCBs and NAEs, with cut-offs for interpretation of very small (Cohen's d< 0.2); small (Cohen's d (0.2-0.5)); medium (Cohen's d (0.5-0.8)); large (Cohen's d (0.8-1.2)); and very large (Cohen's d≥ 1.2). A positive value of the Cohen’s d effect size or the estimate (β, 95%CI) indicates higher values in *APOE*-ɛ4 carriers. | | | | | | |

| Supplementary Table 9. Changes in cardiovascular and lifestyle risk factors by *APOE*-ɛ4 genotype | | | | | | | | | | |
| --- | --- | --- | --- | --- | --- | --- | --- | --- | --- | --- |
|  |  | **APOE-ɛ4 non-carriers (n=83)** | | | **APOE-ɛ4 carriers (n=19)** | | | **Differences (Ref = APOE-ɛ4 non-carriers)** | | |
| **Compound** | **Time** | **Mean change (95%CI)** | **Cohen's d** | **P-value^#^** | **Mean change (95%CI)** | **Cohen's d** | **P-value^#^** | **Cohen's d** | **β (95% CI)^*^** | **P-value^*^** |
| MedDiet adherence | 6 months | 4.0 (3.3, 4.6) | 1.47 | **<0.001** | 3.8 (2.1, 5.6) | 1.56 | **<0.001** | -0.08 | -0.71 (-2.07, 0.65) | 0.311 |
|  | 1 year | 3.4 (2.7, 4.1) | 1.26 | **<0.001** | 4.7 (2.8, 6.7) | 1.74 | **<0.001** | 0.73 | 0.99 (-0.46, 2.45) | 0.184 |
|  | 3 years | 3.0 (2.3, 3.7) | 1.12 | **<0.001** | 3.5 (1.8, 5.2) | 1.32 | **<0.001** | 0.28 | -0.03 (-1.55, 1.49) | 0.965 |
| Physical activity (MET x min/week) | 6 months | 715.4 (247.2, 1183.5) | 0.33 | **0.003** | 317.4 (-510.4, 1145.1) | 0.19 | 0.431 | -8.75 | -550.4 (-1492.7, 391.8) | 0.255 |
|  | 1 year | 668.4 (195.4, 1141.4) | 0.30 | **0.006** | 577.4 (-213.2, 1367.9) | 0.33 | 0.142 | -2.00 | -52.2 (-1026.0, 921.6) | 0.917 |
|  | 3 years | 331.1 (-251.0, 913.3) | 0.20 | 0.181 | 329.0 (-1035.4, 1693.3) | 0.09 | 0.623 | -0.04 | -326.3 (-1504.3, 851.6) | 0.589 |
| Weight (kg) | 6 months | -6.2 (-7.3, -5.2) | -0.43 | **<0.001** | -6.7 (-8.7, -4.6) | -0.52 | **<0.001** | -0.20 | 0.35 (-1.93, 2.64) | 0.762 |
|  | 1 year | -5.9 (-7.1, -4.8) | -0.40 | **<0.001** | -6.9 (-9.4, -4.3) | -0.54 | **<0.001** | -0.41 | -0.33 (-2.91, 2.26) | 0.804 |
|  | 3 years | -6.0 (-7.3, -4.7) | -0.38 | **<0.001** | -6.9 (-9.7, -4.2) | -0.55 | **<0.001** | -0.38 | -0.56 (-3.50, 2.39) | 0.711 |
| BMI (kg/m2) | 6 months | -2.3 (-2.7, -1.9) | -0.65 | **<0.001** | -2.4 (-3.1, -1.7) | -0.79 | **<0.001** | -0.06 | 0.17 (-0.70, 1.03) | 0.704 |
|  | 1 year | -2.2 (-2.7, -1.8) | -0.62 | **<0.001** | -2.4 (-3.3, -1.6) | -0.80 | **<0.001** | -0.15 | -0.03 (-0.99, 0.94) | 0.958 |
|  | 3 years | -2.3 (-2.8, -1.8) | -0.61 | **<0.001** | -2.5 (-3.4, -1.7) | -0.85 | **<0.001** | -0.16 | -0.14 (-1.22, 0.94) | 0.801 |
| Hip (cm) | 6 months | -3.8 (-4.5, -3.0) | -0.41 | **<0.001** | -4.1 (-6.0, -2.3) | -0.57 | **<0.001** | -0.17 | -0.08 (-1.84, 1.68) | 0.927 |
|  | 1 year | -3.6 (-4.6, -2.6) | -0.38 | **<0.001** | -4.4 (-6.6, -2.1) | -0.57 | **<0.001** | -0.36 | -0.68 (-2.92, 1.57) | 0.556 |
|  | 3 years | -3.9 (-5.0, -2.7) | -0.41 | **<0.001** | -4.7 (-7.2, -2.1) | -0.56 | **0.002** | -0.37 | -1.01 (-3.66, 1.64) | 0.457 |
| Waist (cm) | 6 months | -6.1 (-7.1, -5.0) | -0.61 | **<0.001** | -5.7 (-7.7, -3.7) | -0.64 | **<0.001** | 0.19 | 1.17 (-1.08, 3.42) | 0.309 |
|  | 1 year | -6.0 (-7.2, -4.8) | -0.59 | **<0.001** | -5.9 (-8.5, -3.3) | -0.78 | **<0.001** | 0.04 | 0.44 (-2.26, 3.14) | 0.750 |
|  | 3 years | -5.7 (-7.1, -4.3) | -0.52 | **<0.001** | -6.4 (-10.2, -2.5) | -0.82 | **0.002** | -0.29 | -0.57 (-3.94, 2.80) | 0.741 |
| Systolic BP (mmHg) | 6 months | -6.3 (-9.6, -3.0) | -0.39 | **<0.001** | -8.9 (-15.6, -2.1) | -0.75 | **0.009** | -0.67 | -3.17 (-11.07, 4.72) | 0.433 |
|  | 1 year | -5.2 (-8.3, -2.2) | -0.40 | **<0.001** | -8.2 (-17.1, 0.7) | -0.65 | **0.042** | -0.78 | -3.62 (-10.81, 3.58) | 0.327 |
|  | 3 years | -7.5 (-11.2, -3.7) | -0.45 | **<0.001** | -11.4 (-19.1, -3.7) | -0.99 | **0.004** | -0.99 | -5.93 (-14.23, 2.38) | 0.166 |
| Diastolic BP (mmHg) | 6 months | -3.5 (-6.1, -1.0) | -0.29 | **0.013** | -9.1 (-14.2, -4.0) | -1.04 | **<0.001** | -1.64 | -5.61 (-11.22, 0.01) | 0.053 |
|  | 1 year | -3.6 (-6.1, -1.1) | -0.33 | **0.005** | -8.0 (-13.4, -2.6) | -0.74 | **0.007** | -1.31 | -3.72 (-9.12, 1.68) | 0.180 |
|  | 3 years | -3.7 (-6.4, -1.0) | -0.31 | **0.009** | -9.6 (-16.3, -3.0) | -0.96 | **0.005** | -1.71 | -5.28 (-11.09, 0.54) | 0.079 |
| HDL-c (mg/dL) | 6 months | 2.9 (1.3, 4.5) | 0.29 | **<0.001** | 4.3 (1.8, 6.7) | 0.36 | **0.002** | 0.51 | 0.77 (-2.78, 4.32) | 0.671 |
|  | 1 year | 3.1 (1.4, 4.8) | 0.34 | **<0.001** | 3.2 (0.2, 6.3) | 0.37 | **0.033** | 0.06 | -0.37 (-3.89, 3.14) | 0.837 |
|  | 3 years | 1.1 (-0.7, 2.9) | 0.11 | 0.217 | 0.3 (-2.5, 3.1) | 0.21 | 0.675 | -0.30 | -1.37 (-5.52, 2.78) | 0.519 |
| LDL-c (mg/dL) | 6 months | -11.8 (-17.9, -5.8) | -0.35 | **<0.001** | -9.0 (-21.8, 3.8) | -0.28 | 0.157 | 0.54 | 2.06 (-11.12, 15.23) | 0.761 |
|  | 1 year | 2.8 (-5.4, 11.0) | 0.06 | 0.554 | -10.6 (-24.7, 3.5) | -0.36 | 0.134 | -2.25 | -16.58 (-32.80, -0.37) | 0.048 |
|  | 3 years | -12.8 (-23.5, -2.2) | -0.30 | **0.035** | -19.6 (-41.5, 2.2) | -0.69 | 0.055 | -1.01 | -10.52 (-30.56, 9.53) | 0.307 |
| Total cholesterol (mg/dL) | 6 months | -11.0 (-18.0, -4.1) | -0.26 | **0.002** | -7.9 (-19.8, 4.0) | -0.24 | 0.179 | 0.57 | 1.85 (-13.08, 16.78) | 0.809 |
|  | 1 year | 3.7 (-5.0, 12.5) | 0.08 | 0.425 | -9.0 (-23.7, 5.7) | -0.18 | 0.249 | -2.09 | -17.95 (-35.29, -0.61) | **0.045** |
|  | 3 years | -14.8 (-25.9, -3.7) | -0.35 | **0.010** | -22.0 (-45.4, 1.4) | -0.56 | 0.054 | -1.06 | -9.89 (-32.21, 12.42) | 0.387 |
| Triglycerides (mg/dL) | 6 months | -24.2 (-37.1, -11.4) | -0.34 | **<0.001** | -32.7 (-69.1, 3.7) | -0.31 | **0.076** | -1.08 | 1.89 (-20.80, 24.59) | 0.870 |
|  | 1 year | -20.1 (-31.8, -8.3) | -0.30 | **<0.001** | -13.3 (-44.0, 17.4) | -0.37 | **0.288** | 0.92 | 0.35 (-23.61, 24.31) | 0.977 |
|  | 3 years | -31.7 (-48.4, -15.0) | -0.45 | **<0.001** | -13.6 (-40.5, 13.3) | -0.40 | **0.236** | 2.21 | 12.86 (-12.66, 38.39) | 0.326 |
| HbA1c (%) | 6 months | -0.4 (-0.5, -0.3) | -0.40 | **<0.001** | -0.4 (-0.6, -0.2) | -0.42 | **0.001** | 0.02 | 0.13 (-0.12, 0.37) | 0.322 |
|  | 1 year | -0.3 (-0.4, -0.2) | -0.35 | **<0.001** | -0.3 (-0.5, -0.1) | -0.29 | **0.015** | 0.02 | 0.13 (-0.12, 0.39) | 0.299 |
|  | 3 years | -0.2 (-0.4, -0.1) | -0.22 | **0.004** | -0.2 (-0.4, 0.1) | -0.25 | 0.169 | 0.08 | 0.13 (-0.21, 0.47) | 0.452 |
| Glucose (mg/dL) | 6 months | -6.5 (-10.2, -2.9) | -0.21 | **<0.001** | -14.9 (-24.5, -5.3) | -0.53 | **0.004** | -2.01 | -5.23 (-14.18, 3.72) | 0.255 |
|  | 1 year | -6.7 (-9.8, -3.7) | -0.29 | **<0.001** | -6.4 (-20.1, 7.3) | -0.24 | 0.277 | 0.08 | 5.80 (-1.94, 13.55) | 0.146 |
|  | 3 years | -2.6 (-7.7, 2.5) | -0.12 | 0.276 | -7.4 (-23.3, 8.4) | -0.37 | 0.218 | -1.01 | 0.60 (-11.14, 12.34) | 0.920 |
| Insulin (uUI/mL) | 6 months | -1.5 (-2.2, -0.8) | -0.32 | **<0.001** | -1.8 (-3.5, 0.0) | -0.40 | **0.048** | -0.15 | -0.38 (-1.80, 1.03) | 0.594 |
|  | 1 year | -1.5 (-2.3, -0.6) | -0.32 | **<0.001** | -2.2 (-3.9, -0.4) | -0.40 | **0.020** | -0.36 | -0.64 (-2.27, 0.98) | 0.440 |
|  | 3 years | -2.4 (-3.2, -1.5) | -0.47 | **<0.001** | -3.0 (-5.1, -0.9) | -0.57 | **0.012** | -0.32 | -0.55 (-2.22, 1.12) | 0.519 |
| HOMA-IR | 6 months | -0.5 (-0.8, -0.3) | -0.33 | **<0.001** | -0.9 (-1.6, -0.1) | -0.46 | **0.026** | -0.29 | -0.19 (-0.71, 0.33) | 0.475 |
|  | 1 year | -0.5 (-0.8, -0.3) | -0.33 | **<0.001** | -0.8 (-1.7, 0.0) | -0.35 | **0.043** | -0.27 | -0.08 (-0.65, 0.48) | 0.773 |
|  | 3 years | -0.7 (-1.0, -0.3) | -0.37 | **<0.001** | -1.2 (-2.3, -0.2) | -0.58 | **0.031** | -0.47 | -0.33 (-1.03, 0.38) | 0.367 |
| The effect size of changes within group (*APOE*-ɛ4 carriers and non-carriers), and of differences in the rate of change between groups (*APOE* genotypes), was estimated with the Cohen’s d, with cut-offs for interpretation of very small (Cohen's d< 0.2); small (Cohen's d (0.2-0.5)); medium (Cohen's d (0.5-0.8)); large (Cohen's d (0.8-1.2)); and very large (Cohen's d≥ 1.2). A positive value of the Cohen’s d effect size of *APOE* differences indicates the effect is in favor of *APOE*- ɛ4 carriers. ^#^Linear mixed effects models were used to analyze within-group changes, and were adjusted by gender, age, smoking status and use of lipid-lowering agents. ^*^ANCOVA models were used to analyze between group differences in the rate of change with respect to baseline. These models used as outcome variables the changes from baseline to 6 months, 1 year or 3 years, and included *APOE* genotype as an independent variable adjusting for the baseline score, gender, age, smoking status and use of lipid-lowering agents. A positive value of modeled differences (β, 95%CI) indicates the effect is in favor of *APOE*-ɛ4 carriers. Bold values denote statistical significance at the p < 0.05 level. | | | | | | | | | | |
